# Genome sequence of *Hydrangea macrophylla* and its application in analysis of the double flower phenotype

**DOI:** 10.1093/dnares/dsaa026

**Published:** 2020-11-11

**Authors:** Kenji Nashima, Kenta Shirasawa, Andrea Ghelfi, Hideki Hirakawa, Sachiko Isobe, Takuro Suyama, Takuya Wada, Takeshi Kurokura, Tatuya Uemachi, Mirai Azuma, Midori Akutsu, Masaharu Kodama, Yoshiko Nakazawa, Kiyoshi Namai

**Affiliations:** 1 Department of Bioagricultural Sciences, College of Bioresource Sciences, Nihon University, Fujisawa, Kanagawa 252-0880, Japan; 2 Kazusa DNA Research Institute, Kisarazu, Chiba 292-0813Japan; 3 Fukuoka Agriculture and Forestry Research Center, Chikushino, Fukuoka 818-8549, Japan; 4 Department of Agrobiology and Bioresources, Faculty of Agriculture, Utsunomiya University, Utsunomiya, Tochigi 321-8505, Japan; 5 Department of Biological Resources Management, School of Environmental Science, University of Shiga Prefecture, Hikone, Shiga 522-0057, Japan; 6 Tochigi Prefectural Agricultural Experimental Station, Utsunomiya, Tochigi 320-0002, Japan

**Keywords:** hydrangea, double flower, *de novo* genome sequencing, DNA marker

## Abstract

Owing to its high ornamental value, the double flower phenotype of hydrangea (*Hydrangea macrophylla*) is one of its most important traits. In this study, genome sequence information was obtained to explore effective DNA markers and the causative genes for double flower production in hydrangea. Single-molecule real-time sequencing data followed by a Hi-C analysis were employed. Two haplotype-phased sequences were obtained from the heterozygous genome of hydrangea. One assembly consisted of 3,779 scaffolds (2.256 Gb in length and N50 of 1.5 Mb), the other also contained 3,779 scaffolds (2.227 Gb in length, and N50 of 1.4 Mb). A total of 36,930 genes were predicted in the sequences, of which 32,205 and 32,222 were found in each haplotype. A pair of 18 pseudomolecules was constructed along with a high-density single-nucleotide polymorphism (SNP) genetic linkage map. Using the genome sequence data, and two F_2_ populations, the SNPs linked to double flower loci (*d_jo_* and *d_su_*) were discovered. DNA markers linked to *d_jo_* and *d_su_* were developed, and these could distinguish the recessive double flower allele for each locus, respectively. The *LEAFY* gene is a very likely candidate as the causative gene for *d_su_*_,_ since frameshift was specifically observed in the double flower accession with *d_su_*.

## 1. Introduction

*Hydrangea macrophylla* (Thunb.) Ser., commonly known as hydrangea, originated in Japan, and since it is the place of origin, there are rich genetic resources for this species there. Wild hydrangea accessions with superior characteristics have been used as breeding parents to create attractive cultivars, and it has a long history of use as an ornamental garden plant in temperate regions. In hydrangea, there are both decorative and non-decorative flowers within groups of flowers on the stems of the Hydrangea inflorescence. Decorative flowers have large ornamental petaloid sepals that attract pollinators, whereas non-decorative flowers have inconspicuous perianths with normal sepals that instead of play a major role in seed production.^1–3^ In hydrangea, there are two decorative flower phenotypes: a single flower and double flower. Single flowers generally have four petaloid sepals per decorative flower, while in double flowers, it is approximately fourteen.^4^ Double flowers do not have stamens or petals in the decorative flower.^4^ Therefore, petals and stamens could be converted to petaloid sepals since the number of petaloid sepals increases, and stamens and petals are lost. Because of their high ornamental value, the production of double flowers is an important breeding target in hydrangea cultivation.

**Figure 1:**
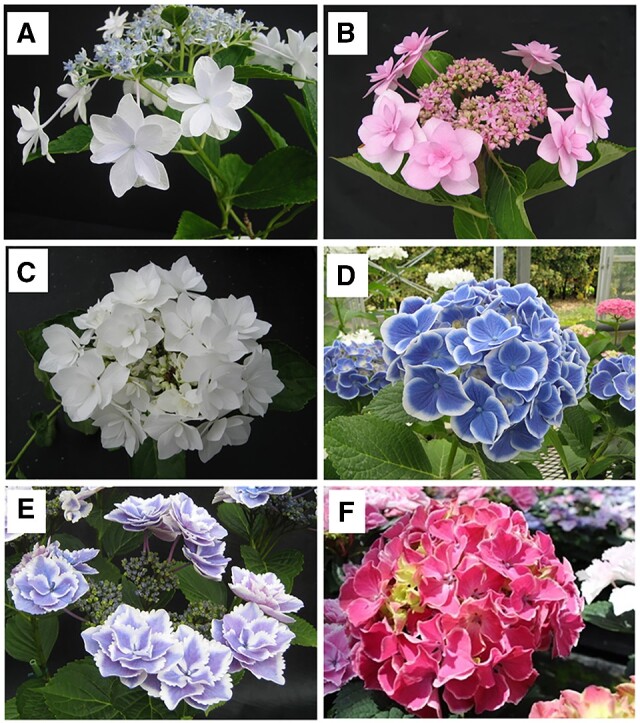
Flower phenotypes of hydrangea accessions. A: ‘Sumidanohanabi’ (double flower). B: ‘Jogasaki’ (double flower). C: ‘Posy Bouquet Grace’ (double flower). D: ‘Blue Picotee Manaslu’ (single flower). E: ‘Kirakiraboshi’ (double flower). F: ‘Frau Yoshimi’ (single flower).

The double flower cultivars ‘Sumidanohanabi’ (Fig. 1A) and ‘Jogasaki’ (Fig. 1B) have been used as breeding parents in Japan^4^ to obtain double flower progenies. Both cultivars lack stamens and petals and show increased petaloid sepals in their decorative flowers compared with single flower cultivars (Supplementary Fig. S1A–C). For non-decorative flowers, while they lack stamen similar to decorative flowers, slightly sepaloid petals are observed which increased its number (Supplementary Fig. S1D–F). Sepals in non-decorative flowers are observed in both single and double flower accessions (Supplementary Fig. S1G and H). Because both double flower cultivars lack stamen, they could not be used as pollen parents. However, these cultivars still have fertile pistils, they could be used as seed parent. Previous studies have suggested that the double flower phenotype is a recessive trait controlled by a single major gene.^4^^,^^5^ Suyama et al.^4^ found that crosses between the progeny of ‘Sumidanohanabi’ and the progeny of ‘Jogasaki’ produced only single flower descendants. Thus, it was also suggested that the genes controlling the double flower phenotype are different.^4^ We named the double flower locus *d_su_* as the locus controlling the double flower phenotype of ‘Sumidanohanabi’ and the double flower locus *d_jo_* as the locus controlling the double flower phenotype of ‘Jogasaki.’ Waki et al.^5^ identified *d_su_* on the genetic linkage map. They also found that the DNA marker STAB045 was the closest marker to *d_su_* and that STAB045 could help distinguish flower phenotypes with a high degree of agreement. Contrarily, *d_jo_* has not been identified, and the DNA marker linked to *d_jo_* has not been developed. It is still not known whether *d_jo_* and *d_su_* are at the same loci.

The mechanisms and genes controlling the double flower phenotype in hydrangea have not been clarified. Waki et al.^5^ hypothesized that the mutation of C-class genes could be associated with the double flower phenotype of ‘Sumidanohanabi’ since the C-class gene mutant of *Arabidopsis thaliana* and C-class gene-repressed petunias produce double flowers.^6^ However, the double flower phenotype of hydrangea is morphologically different from that of *Arabidopsis thaliana* and petunia: petals and stamens converted to petaloid sepals, while stamens converted to petals in *Arabidopsis thaiana* and petunia. This suggests that the genes controlling double flower production in hydrangea differ from genes controlling double flower production in other plant species. Identification of the genes controlling double flower production in hydrangea could reveal novel regulatory mechanisms of flower development.

Genomic information is essential for DNA marker development and identification of genes controlling specific phenotypes. However, no reference genome sequence is publicly available for hydrangea so far. Although a genome assembly of hydrangea (1.6 Gb) using only short-read data has been reported,^7^ the resultant assembly is so fragmented that it comprises 1,519,429 contigs with an N50 size 2,447 bp, and has not been disclosed. Improved, advanced long-read technologies and bioinformatics methods would make it possible to determine the sequences of complex genomes. An assembly strategy for single-molecule real-time sequencing data followed by a Hi-C analysis has been developed to generate haplotype-phased sequences in heterozygous regions of diploid genomes.^8^ Genome sequences at the chromosome level could be obtained with a Hi-C clustering analysis^9^ and a genetic linkage analysis.^10^ Such a genomic sequence could provide basic information to identify genes and DNA markers of interest and to discover allelic sequence variations. In this study, we constructed the genomic DNA sequence, obtained single-nucleotide polymorphisms (SNPs) information, and performed gene prediction. We also developed DNA markers linked to *d_jo_* using SNP information obtained by double digest restriction site associated DNA sequence (ddRAD-Seq) analysis of the F_2_ mapping population 12GM1, which segregated double flower phenotypes of *d_jo_*. In addition, we attempted to identify the causative genes for *d_jo_* and *d_su_*.

## 2. Materials and methods

### 2.1. *De novo* assembly of the hydrangea genome

For genomic DNA sequencing, *H. macrophylla* ‘Aogashima-1,’ collected from Aogashima Island of the Izu Islands in the Tokyo Prefecture, Japan, was used. Genomic DNA was extracted from the young leaves using Genomic-Tip (Qiagen, Hilden, Germany). First, we constructed a sequencing library (insert size 500 bp) with TruSeq DNA PCR-Free Library Prep Kit (Illumina, San Diego, CA, USA) to sequence on HiSeqX (Illumina). The size of the ‘Aogashima-1’ genome was estimated using Jellyfish v2.1.4.^11^ After removing the adapter sequences and trimming low-quality reads, high-quality reads were assembled using Platanus.^12^ The resultant sequences were designated HMA_r0.1. The completeness of the assembly was assessed with sets of BUSCO v.1.1b.^13^

Next, an PacBio single molecule, real-Time (SMRT) library was constructed with the SMRTbell Express Template Prep Kit 2.0 (PacBio, Menlo Park, CA, USA) in accordance with the manufacturer’s protocol and sequenced using SMRT Cell v2.1 on a Sequel System. The sequence reads were assembled using FALCON v.1.8.8^14^ to generate primary contig sequences and associate contigs representing alternative alleles. Haplotype-resolved assemblies (i.e. haplotigs) were generated using FALCON-Unzip v.1.8.8.^14^ Potential sequence errors in the contigs were corrected twice with ARROW v.2.2.1 implemented in SMRT Link v.5.0 (PacBio) followed by one polishing with Pilon.^15^ The resultant sequences were designated HMA_r1.0. Subsequently, a Hi-C library was constructed with Proximo Hi-C (Plant) Kit (Phase Genomics, Seattle, WA, USA) and sequenced on HiSeqX (Illumina). After removing the adapter sequences and trimming the low-quality reads, high-quality Hi-C reads were used to generate two haplotype-phased sequences (Phase 0 and Phase 1) from the primary contigs and haplotig sequences with FALCON-Phase.^8^ The resultant sequences were designated HMA_r1.1. Haplotype phase 0 was derived from one of a pair of homologous chromosomes of ‘Aogashima-1’, and the haplotype phase 1 was derived from other ‘Aogashima-1’ chromosomes.

To validate the accuracy of the sequences, we developed a genetic map based on SNPs, from a ddRAD-Seq analysis on the 12GM1, F_2_ mapping population (*n* = 147), maintained at the Fukuoka Agriculture and Forestry Research Center, Japan. The 12GM1 population was generated from a cross between ‘Posy Bouquet Grace’ (Fig. 1C) and ‘Blue Picotee Manaslu’ (Fig. 1D). Genomic DNA was extracted from the leaves with a DNeasy Plant Mini Kit (Qiagen). A ddRAD-Seq library was constructed as described by Shirasawa et al.^16^ and sequenced with HiSeq4000. Sequence reads were processed as described by Shirasawa et al.^16^ and mapped on the HMA_r1.1 as a reference. From the mapping alignment, high-confidence biallelic SNPs were obtained with the following filtering options: –minDP 5 –minQ 10 –max-missing 0.5. The genetic map was constructed using Lep-Map3.^17^

Potential mis-jointed points in Phase 0 and 1 sequences of HMA_r1.1 were cut and re-joined, based on the marker order in the genetic map. The resultant sequences were named HMA_r1.2. Haplotype-phased pseudomolecule sequences were generated based on HMA_r1.2 and genetic map using ALLMAPS.^18^ The resultant sequences were named HMA_r1.2.pmol, as two haplotype-phased pseudomolecule sequences of the ‘Aogashima-1’ genome. Sequences that were unassigned to the genetic map were connected and termed chromosome 0. The constructed pseudomolecule sequence was corresponded with the previously reported SSR marker-based genetic linkage map^5^ by a BLAST search of simple sequence repeat (SSR) marker sequences. SSR markers that were included in the hydrangea genetic linkage map^5^ were searched for HMA_r1.2.pmol. When more than two SSR markers in a linkage group were located on the same pseudomolecule, the pseudomolecule was treated as corresponded to the genetic linkage map. The nomenclature of the pseudomolecule number was assigned in accordance with linkage group number of the genetic map.^5^

### 2.2. Gene prediction

For gene prediction, we performed an Iso-Seq analysis. Total RNA was extracted from 12 samples of ‘Aogashima-1’: flower buds (2 stages); decorative flowers (2 stages); coloured and colourless non-decorative flowers; fruits; stem; roots; leaf buds and one-day light-intercepted leaves and buds. In addition, the mixed sample (Sample No. 29) listed in Supplementary Table S1 was included. Iso-Seq libraries were prepared following the manufacture’s Iso-Seq Express Template Preparation (PacBio) protocol and sequenced on a Sequel System (PacBio). The raw reads obtained were treated with ISO-Seq3 pipeline, implemented in SMRT Link v.5.0 (PacBio) to generate full-length, high-quality consensus isoforms. In parallel, RNA-Seq data were also obtained from the 16 samples listed in Supplementary Table S1. Total RNA extracted from the samples was converted into cDNA and sequenced on HiSeq2000, Hiseq2500 (Illumina), and NovaSeq6000 (Illumina). The Iso-Seq isoform sequences and the RNA-Seq short-reads were employed for gene prediction.

We used *ab-initio*-, evidence-, and homology-based gene prediction methods to identify putative protein-encoding genes in the genome assemblies. For this prediction, unigene sets generated from (i) the Iso-Seq isoforms; (ii) *de novo* assembly of the RNA-Seq short-reads with Trinity-v2.4.0^19^; (iii) peptide sequences predicted from the genomes of *Arabidopsis thaliana*, *Arachis hypogaea*, *Cannabis sativa*, *Capsicum annuum*, *Cucumis sativus*, *Populus trichocarpa*, and *Quercus lobata*; and (iv) *ab-initio* genes, were predicted with Augustus-v3.3.1.^20^ The unigene sequences were aligned onto the genome assembly with BLAT,^21^ and the genome positions of the genes were listed in general feature format version 3 with blat2gff.pl (https://github.com/vikas0633/perl/blob/master/blat2gff.pl)(12 November 2020, date last accessed). Gene annotation was performed with Hayai-annotation Plants.^22^ Completeness of the gene prediction was assessed with sets of BUSCO v4.0.6.^13^

### 2.3. Detection of SNPs linked to double flower phenotype

For identification of SNPs linked to double flower loci *d_jo_* and *d_su_*, ddRAD-Seq data analysis was performed. ddRAD-Seq data of the 12GM1 population described above were used to identify *d_jo_*. For identification of SNPs linked to double flower locus *d_su_*, KF population^5^—93 F_2_ individuals of ‘Kirakiraboshi’ (Fig. 1E) and ‘Frau Yoshimi’ (Fig. 1F)—were used for ddRAD-Seq analysis. The KF population was maintained at the Tochigi Prefectural Agricultural Experimental Station, Japan. ddRAD-Seq analysis of the KF population was performed using the same method used for the 12GM1 population.

ddRAD-Seq data of the 12GM1 and KF populations were processed as follows: low-quality sequences were removed, and adapters were trimmed using Trimmomatic-0.36^23^ (LEADING : 10, TRAILING : 10, SLIDING WINDOW: 4:15, MINLEN : 51). BWA-MEM (v 0.7.15-r1140) was used for mapping to the genome sequence. The resultant sequence alignment/map format files were converted to binary sequence alignment/map format files and subjected to SNP calling using the mpileup option of SAMtools^24^ (v 1.4.1) and the view option of BCFtools (parameter -vcg). If the DP of called SNP in individuals was under 5%, the genotype was treated as missing. SNPs with 5% or more of missing genotype were filtered out. Each SNP was evaluated for its degree of agreement with the flower phenotype. The degree of agreement with the flower phenotype was calculated as the percentage of individuals agreeing with the model (double flower phenotype is observed when homozygous of allele is from the double flower parent, while single flower phenotype is observed when heterozygous or homozygous of allele is from the single flower parent) among the population.

### 2.4. DNA marker development and analysis for *d_jo_*

Cleaved amplified polymorphic sequence (CAPS) markers were designed based on SNPs that were completely co-segregated to the double flower locus *d_jo_*. Primers were designed using Primer3^25^ under conditions with product size ranging from 150 to 350 bp, primer size from 18 to 27 bp, and primer TM from 57 to 63°C. PCR assays were conducted in a total volume of 10 μL, containing 5 μL of GoTaq Master Mix (Promega, Madison, WI, USA), 1 mM each of forward and reverse primer, and 5 ng of template DNA. The PCR conditions were 94°C for 2 min, 35 cycles of denaturation at 94°C for 1 min, annealing at 55°C for 1 min, and extension at 72°C for 1 min; and a final extension step at 72°C for 3 min. Subsequently, restriction enzyme assay was conducted in a total volume of 10 μL, containing 5 μL PCR product and 10 units of restriction enzyme. The restriction assay product was stained with 1× GRRED (Biocraft, Tokyo, Japan) and separated in 1.5% (w/v) agarose gel in Tris-acetate-EDTA (TAE) buffer. The CAPS marker, whose genotype was coincident with the SNP genotype in the 12GM1 population, was selected as the relevant marker. The relevant CAPS marker named J01 was designed from SNP on scaffold : 0008 F-2, position: 780104. The primer sequences of J01 were: Forward: 5′-CTGGCAGATTCCTCCTGAC-3′ and Reverse: 5′-TATTTCCTTGGGGAGGCTCT-3′. A restriction enzyme assay was performed at 65°C for 3 h using *Taq* I (New England Biolabs, Ipswich, MA, USA). CAPS marker J01 was applied to the 14GT77 population (64 F_2_ individuals of ‘Posy Bouquet Grace’ × ‘Chibori’) and the 15IJP1 population (98 F_1_ individuals of ‘Izunohana’ × 03JP1) in addition to the 12GM1 population that segregate the double flower locus *d_jo_*.

### 2.5. Resequencing and comparison of LEAFY gene sequence and DNA marker development

We performed resequencing of genomic DNA to compare sequences for accessions of ‘Kirakiraboshi,’ ‘Frau Yoshimi,’ ‘Posy Bouquet Grace,’ and ‘Blue Picotee Manaslu.’ Sequencing libraries (insert size 500 bp) for the four lines were constructed with TruSeq DNA PCR-Free Library Prep Kit (Illumina) to sequence on a HiSeqX (Illumina). From the sequence reads obtained, low-quality bases were deleted with PRINSEQ v0.20.4,^26^ and adaptor sequences were trimmed with fastx clipper (parameter, ‐a AGATCGGAAGAGC) in FASTX-Toolkit v. 0.0.13 (http://hannonlab.cshl.edu/fastx_toolkit)(12 November 2020, date last accessed). High-quality reads were aligned on HMA_r1.2 with Bowtie2^27^ v. 2.2.3 to detect sequence variant candidates using the mpileup command in SAMtools v 0.1.19.^24^ High-confidence variants were selected using VCFtools^28^ v. 0.1.12b with parameters of –minDP 10, –maxDP 100, –minQ 999, –max-missing 1.

For comparison of the *LEAFY (LFY)* sequence in ‘Kirakiraboshi,’ ‘Frau Yoshimi,’ ‘Posy Bouquet Grace,’ and ‘Blue Picotee Manaslu,’ BLAST analysis was performed to confirm detected sequence variants using the genomic sequence of *LFY* (Scaffold 0577 F, position 678200-684639) as query, and the genomic DNA sequence of each cultivar as the database. These data analyses were performed using CLC main workbench (Qiagen). INDEL marker S01 that amplifies the second intron of *LFY*, was designed by visual inspection (Forward: 5′-CATCATTAATAGTGGTGACAG-3′, Reverse: 5′-CACACATGAATTAGTAGCTC-3′). The PCR conditions were 94°C for 2 min, 35 cycles of denaturation at 94°C for 1 min, annealing at 55°C for 1 min, extension at 72°C for 1 min; and a final extension step at 72°C for 3 min. The PCR product was stained with 1x GRRED (Biocraft) and separated in 2.5% (w/v) agarose gel in TAE buffer.

### 2.6. Cloning and sequence determination of *LFY* gene of ‘Kirakiraboshi’ and ‘Frau Yoshimi’

Total RNA was isolated from flower buds of ‘Kirakiraboshi’ and ‘Frau Yoshimi’ using RNAiso Plus (TaKaRa, Japan), and reverse transcribed using a PrimeScript II 1st strand cDNA Synthesis Kit (TaKaRa, Japan). The sequence of the *LFY* gene was amplified by PCR in a 50-µL reaction mixture using TaKaRa Ex Taq Hot Start Version (TaKaRa Bio, Shiga, Japan) and the *LFY* specific primer (Forward: 5′-ATGGCTCCACTACCTCCACC-3′ and Reverse: 5′-CTAACACCCTCTAAAAGCAG-3′). These PCR products were purified and inserted into a pMD20-T vector using a Mighty TA-cloning kit (TaKaRa Bio). The sequence of the *LFY* coding sequence (CDS) in the pMD20-T vector was analysed using a 3130xl Genetic Analyzer (Applied Biosystems, Foster City, CA, USA) for DNA sequencing. Sequence alignments were obtained using the CLC main workbench (Qiagen).

### 2.7. DNA marker assessment across hydrangea accessions

Thirty-five *H. macrophylla* accessions were used for the assessment of DNA markers for the double flower phenotype. Genotyping for J01 was performed as described above. Genotyping for S01 was performed by fragment analysis as follows. PCR amplification was performed in a 10-μL reaction mixture containing 5 μL of GoTaq Master Mix (Promega), 5 pmol FAM-labeled universal primer (5′- FAM-gctacggactgacctcggac -3′), 2.5 pmol forward primer with universal adapter sequence (5′- gctacggactgacctcggacCATCATTAATAGTGGTGACAG -3′), 5 pmol reverse primer, and 5 ng of template DNA. DNA was amplified in 35 cycles of 94°C for 1 min, 55°C for 1 min, and 72°C for 2 min; and a final extension of 5 min at 72°C. The amplified PCR products were separated and detected with a PRISM 3130xl Genetic Analyzer (Applied Biosystems, USA). The sizes of the amplified bands were scored against internal-standard DNA (400HD-ROX, Applied Biosystems, USA) with GeneMapper software (Applied Biosystems, USA).

## 3. Results and discussion

### 3.1. Draft genome assembly with long-read and Hi-C technologies

The size of the hydrangea genome was estimated by k-mer-distribution analysis with the short-read of 132.3 Gb data. The resultant distribution pattern indicated two peaks, representing homozygous (right peak) and heterozygous (left peak) genomes, respectively (Supplementary Fig. S2). The haploid genome of hydrangea was estimated to be 2.2 Gb in size. The short-reads were assembled into 612,846 scaffold sequences. The total length of the resultant scaffolds, i.e. HMA_r0.1, was 1.7 Gb with an N50 length of 9.1 kb (Supplementary Table S2). Only 72.2% of complete single-copy orthologues in plant genomes were identified by BUSCO analysis (Supplementary Table S2).

Next, we employed long sequence technology to extend the sequence contiguity and to improve the genome coverage. A total of 106.9 Gb of reads (48.6× genome coverage) with an N50 read length of 28.8 kb was obtained from 14 SMRT Cells. The long-reads were assembled, followed by sequence error corrections into 15,791 contigs consisting of 3,779 primary contigs (2.178 Gb in length and N50 of 1.4 Mb), and 12,012 haplotig sequences (1.436 Gb in length and N50 of 184 kb). These resultant sequences were named HMA_r1.0 (Supplementary Table S2). To obtain two haplotype-phased complete-length sequences, 697 M reads of Hi-C data (105.3 Gb) were obtained and subjected to FALCON-Phase. The resultant haplotype-phased sequences, i.e. HMA_r.1.1, consisted of 3,779 scaffolds (2.256 Gb in length and N50 of 1.5 Mb) for Phase 0, and 3,779 scaffolds (2.227 Gb in length, and N50 of 1.4 Mb) for Phase 1 (Supplementary Table S2).

### 3.2. Pseudomolecule sequences based on genetic mapping

To detect potential errors in the assembly and to assign the contig sequences onto the hydrangea chromosomes, we established an F_2_ genetic map based on SNPs derived from ddRAD-Seq analysis. Approximately 1.8 million high-quality ddRAD-Seq reads per sample were obtained from the mapping population and mapped to the Phase 0 and Phase 1 sequences, with alignment rates of 88.4% and 88.7%, respectively. A set of SNPs detected from the alignments were classified into 18 groups and ordered to construct two genetic maps for the two-phased sequences (2,849.3 cM in length with 3,980 SNPs 2,944.5 cM in length with 4,071 SNPs). The phased sequences were aligned on each genetic map to establish haplotype-phased, chromosome-level pseudomolecule sequences. During this process, one contig was cut due to possible mis-assembly and was designated as HMA_r1.2. The resultant pseudomolecule sequence HMA_r1.2.pmol for Phase 0 had 730 contigs with a total length of 1,078 Mb, and the other for Phase 1 had 743 contigs spanning 1,076 Mb. The pseudomolecule was named in accordance with the nomenclature of the previous genetic map based on SSRs.^5^ HMA_r1.2.pmol included 36 pseudomolecule sequences of CHR01 to CHR18 for Phase 0 and Phase 1, which corresponded to the chromosome number of the diploid *H. macrophylla* genome (2*n* = 36).^29^ Correspondence between the pseudomolecule sequence and previous genetic linkage map^5^ is shown in Supplementary Table S3.

### 3.3. Transcriptome analysis followed by gene prediction

In the Iso-Seq analysis Circular Consensus Sequence (CCS) reads were generated from the raw sequence reads. The CCS reads were classified as full-length and non-full-length reads, and the full-length reads were clustered to produce consensus isoforms. In total, 116,634 high-quality isoforms were used for gene prediction, while, in the RNA-Seq analysis, a total of 80.7 Gb reads were obtained and assembled into 12,265 unigenes. The high-quality isoforms and unigenes, together with gene sequences predicted from the *Arabidopsis thaliana*, *Arachis hypogaea*, *Cannabis sativa*, *Capsicum annuum*, *Cucumis sativus*, *Populus trichocarpa*, and *Quercus lobate* genomes were aligned onto the assembly sequence of the hydrangea genome. By adding *ab-initio* on the genes, a total of 36,930 putative protein-encoding genes were predicted in the hydrangea genome, out of which 32,205 and 32,222 genes were found in the Phase 0 and Phase 1 sequences. The 36,930 genes included 89.9% complete BUSCOs.

### 3.4. Identification of SNPs tightly linked to double flower phenotype *d_jo_*

**Figure 2:**
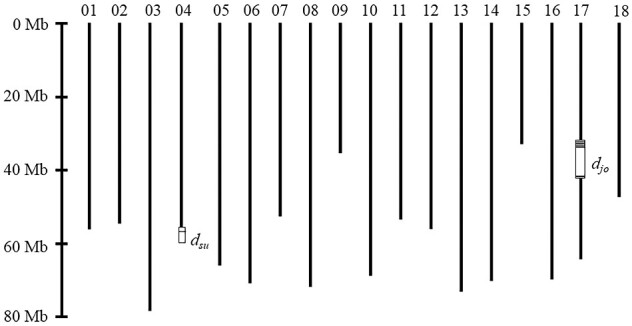
Loci controlling double flower phenotype on the pseudomolecule sequence HMA_r1.2 pmol. Predicted double flower loci *d_su_* and *d_jo_* are shown as boxes. SNPs with more than a 95% agreement with phenotype shown in Tables 1 and 2 are shown as horizontal lines in the boxes. Numbers on the *y*-axis for pseudomolecules indicate the chromosome number for chromosomes CHR01 to CHR18.

To identify SNPs tightly linked to the *d_jo_* derived double flower phenotype of ‘Jogasaki,’ ddRAD-Seq analysis was performed on the 12GM1 population, which segregates the double flower phenotype of ‘Jogasaki.’ According to data of variety under Plant Variety Protection (http://www.hinshu2.maff.go.jp/)(12 November 2020, date last accessed), ‘Posy Bouquet Grace’ was obtained by a cross between ‘Jogasaki’ derived progenies. Therefore, the double flower phenotype of ‘Posy Bouquet Grace’ and its F_2_ population 12GM1 should contain the *d_jo_* mutation. As a result, 14,006 SNPs were called by ddRAD-Seq analysis of the 12GM1 population. In this population, the double flower phenotype was expected when the plant was homozygous for the ‘Posy Bouquet Grace’ genotype, and the single flower phenotype was expected when the plant was homozygous for ‘Blue Picotee Manaslu’ or was heterozygous. Each SNP was tested for its degree of agreement with this model. As a result, nine SNPs were found to have more than a 95% degree of agreement, and six SNPs were completely co-segregated with flower phenotype (Table 1). While these SNPs were located on 33.7 Mb to 43.8 Mb on CHR17, *d_jo_* was suggested to be located at approximately 33.7 Mb to 43.8 Mb on CHR17 (Fig. 2).

**Table 1:** SNPs showing agreement (greater than 95%) with double flower phenotype of djo in 12GM1 population

Position at HMA_r1.2 Phase 0	Position at HMA_r.1.2.pmol Phase 0	Sequence variant		Degree of agreement (%)	Frequency of double flower phenotype (double flower/all)		
		Posy Bouquet Grace	Blue Picotee manasulu		Homozygous of ‘Posy Bouquet Grace’	Heterozygous	Homozygous of ‘Blue Picotee Manasulu’
0008F-2_3250598	CHR17_43855890	A	G	100	37/37	0/61	0/47
0008F-2_3250523	CHR17_43855965	A	C	100	37/37	0/61	0/47
0008F-2_780104	CHR17_43626384	C	A	100	37/37	0/60	0/48
0259F_404610	CHR17_33708714	T	A	100	37/37	0/60	0/48
1207F_365533	CHR17_34478996	C	T	100	38/38	0/61	0/48
1207F_372121	CHR17_34485554	C	A	100	38/38	0/61	0/47
0437F_170787	CHR17_35610977	G	A	97.9	36/37	1/60	1/49
0437F_170821	CHR17_35611011	A	G	97.9	36/37	1/60	1/49
0994F_216439	CHR17_35213652	C	T	97.9	36/37	1/60	1/49

### 3.5. Identification of SNPs tightly linked to double flower phenotype *d_su_*

The KF population that segregates the double flower phenotype derived from ‘Sumidanohanabi’ was used to identify SNPs linked to the double flower phenotype of ‘Sumidanohanabi.’ First, we attempted to find co-segregated scaffolds with the double flower phenotype using ddRAD-Seq analysis of the KF population. As a result of the ddRAD-Seq analysis, 15,102 of SNPs were called. In this population, the double flower phenotype was expected when the plant was homozygous for the ‘Kirakiraboshi’ genotype, and the single flower phenotype was expected when the plant was homozygous for ‘Frau Yoshimi’ or was heterozygous. Each SNP was tested for its degree of agreement with this model. As a result, five SNPs on three scaffolds were found to have more than a 95% degree of agreement with the model (Table 2). Since the SNPs on scaffold 3145 F all had the same genotype across the KF population, three loci—on scaffold 0577 F, 3145 F, 0109 F—were detected. Although the numbers of heterozygous and homozygous of ‘Frau Yoshimi’ were the same (Table 2), recombination was observed in two individuals between 0577 and 3145 F (Table 2). According to genotypes of the KF population, these three loci were tightly linked within 5 cM; 0109 F (0 cM)—3145F (3.9 cM)—0577F (5.0 cM). The scaffold 3145 and 0577 F sequences were not included in the pseudomolecule sequence because no segregated SNPs were detected on 3145 and 0577 F in the 12GM1 population. However, the SNP at position 868,569 in 0109 F was found at position 57,436,162 in CHR04 (the terminal region of CHR04). Thus, these three loci and locus *d_su_*, which control the double flower phenotype of ‘Sumidanohanabi,’ were suggested to be located at the terminal region of CHR04 (Fig. 2). Waki et al.^5^ reported that the *d_su_* locus was located on linkage group KF_4, which corresponded to pseudochromosome CHR04 in this study. Therefore, our results were coincident with the previous report. It has been suggested that genes controlling the double flower phenotype differed between ‘Jogasaki’ and ‘Sumidanohanabi’ based on confirmation of the segregation ratio of crossed progenies.^4^ Our study revealed that the double flower phenotype of ‘Jogasaki’ was controlled by the single *d_jo_* locus on CHR17, and the double flower phenotype of ‘Sumidanohanabi’ was controlled by the single *d_su_* locus on CHR04. It is confirmed that genes controlling the double flowers *d_jo_* and *d_su_* are different as suggested by Suyama et al^4^.

**Table 2:** SNPs showing agreement (greater than 95%) with double flower phenotype of *d_su_* in KF population

Position at HMA_r1.2 Phase 0	Position at HMA_r.1.2.pmol Phase 0	Sequence variant		Degree of agreement (%)	Frequency of double flower phenotype (double flower/all)		
		Kirakiraboshi	Frau Yoshimi		Homozygous of ‘Kirakiraboshi’	Heterozygous	Homozygous of ‘Frau Yoshimi’
0577F_1204837	Not assigned	AG	AAACATG	98.9	22/22	0/51	1/20
3145F_55089	Not assigned	TA	TAA	98.9	22/22	0/51	1/20
3145F_55109	Not assigned	G	A	98.9	22/22	0/51	1/20
3145F_55446	Not assigned	G	A	98.9	22/22	0/51	1/20
0109F_868569	CHR04_57436162	C	G	95.7	22/25	0/44	1/24

### 3.6. Prediction of genes controlling double flower for *d_su_* and *d_jo_* from genome sequence

**Figure 3:**
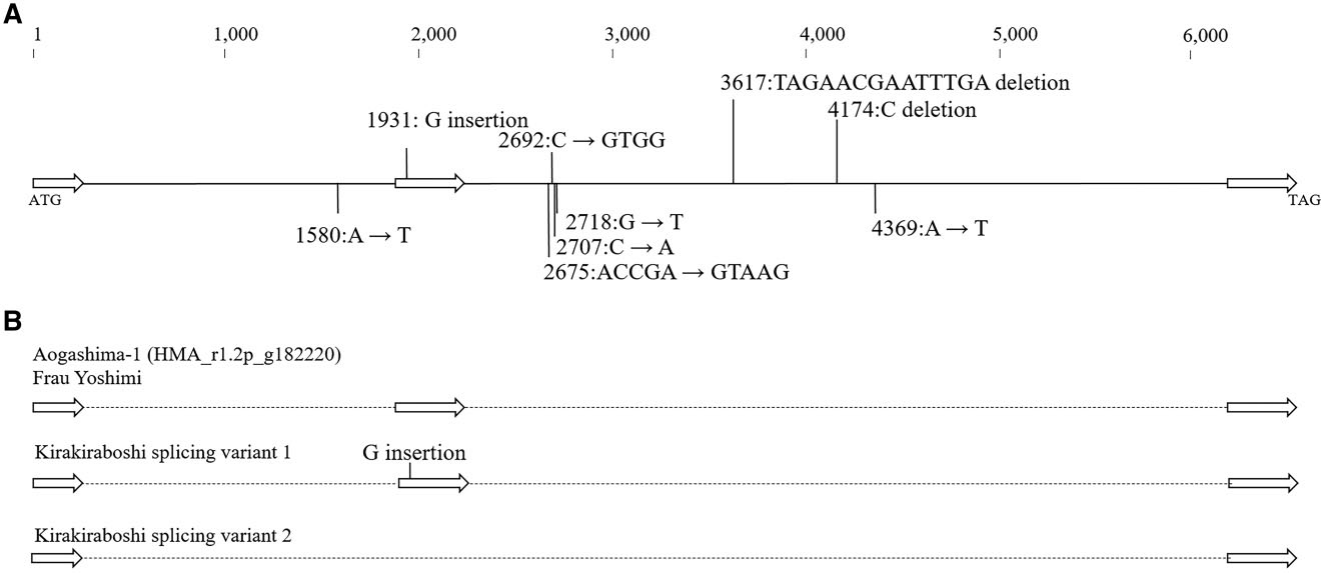
*LFY* sequence polymorphisms observed specifically in ‘Kirakiraboshi’ genomic sequence. A: Polymorphisms observed in genomic sequence of *LFY*. The sequence starts from the initiation codon (ATG) at 678,200 to the termination signal (TAG) at 684,639 in Phase 1 sequence of 0577F of HMA_r1.2. White arrows indicate coding sequences, Exon 1: 1 to 454 bp, Exon 2: 1,888 to 2,255 bp, Exon 3: 6,078 to 6,440 bp. Genetic variants are shown from HMA_r1.2 sequence to ‘Kirakiraboshi.’ B: Observed splicing variants of ‘Kirakiraboshi’. Splicing variant 1 occurred insertion in second exon. Splicing variant 2 occurred loss of second exon.

To find the gene controlling *d_su_* and *d_jo_*, we searched the homeotic genes on the scaffolds shown in Tables 1 and 2. We did not find any notable homeotic gene controlling flower phenotype for *d_jo_*. However, for *d_su_*, the g182220 gene that encoded the homeotic *LFY* gene was found on scaffold 0577 F. To investigate the possibility that it was the causative gene for *d_su_*, variants on *LFY* genomic sequence were searched to identify the ‘Kirakiraboshi’ specific mutation, using the resequencing data of ‘Kirakiraboshi,’ ‘Frau Yoshimi,’ ‘Posy Bouquet Grace,’ and ‘Blue Picotee Manaslu.’ As a result, four INDELs and five sequence variants were ‘Kirakiraboshi’ specific mutations (Fig. 3A).

**Figure 4:**
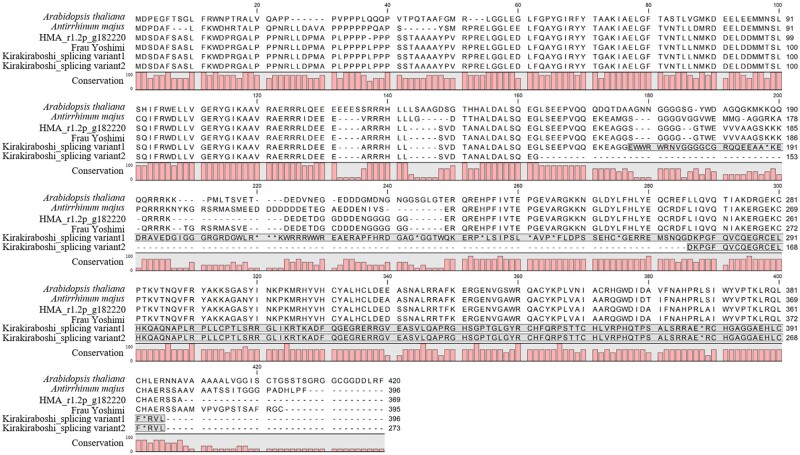
Alignment of LFY protein sequences. Amino acids with grey background show frameshifted regions. Splicing variant was observed, and both sequences showed frameshift in ‘Kirakiraboshi.’ *Arabidopsis thaliana:* ABE66271.1 *Antirrhinum majus*: AAA62574.1.

Cloning and sequencing of the *LFY* CDS were performed on ‘Kirakiraboshi’ and ‘Frau Yoshimi.’ From ‘Frau Yoshimi,’ a single CDS comprising three exons was obtained. From ‘Kirakiraboshi,’ two CDSs with splice variants were obtained. While splicing variant 1 resulted in three exons, splicing variant 2 resulted in only two exons, corresponding to the first and third splice products of splicing variant 1 (Fig. 3B, Supplementary Fig. S3). The deduced amino acid sequences were aligned using the CDSs of ‘Frau Yoshimi’ and ‘Kirakiraboshi,’ g182220 sequence, protein LFY of *Arabidopsis thaliana,* and protein FLO of *Antirrhinum majus*. While the deduced amino acid sequences of ‘Frau Yoshimi’ and g182220 showed sequence similarity in the entire region, frameshift occurred in the two splicing variants obtained from ‘Kirakiraboshi’ and the resulting products had no sequence similarity across the latter half (Fig. 4). The frameshift observed in splicing variant 1 was due to one basepair of DNA insertion in the second exon, at position 1,931 (Fig. 3A, Supplementary Fig. S3). Contrarily, the frameshift observed in splicing variant 2 was due to the complete loss of exon 2 (Fig. 3B, Supplementary Fig. S3).

### 3.7. *LFY* gene function in double flower of hydrangea

In the genomic sequence of ‘Kirakiraboshi,’ an insertion was detected in the second exon of the *LFY* gene. This insertion resulted in a frameshift of cloned mRNA in splicing variant 1. For splicing variant 2, the lack of a second exon resulted in frameshift. Because frameshift occurred in both splicing variants of ‘Kirakiraboshi,’ it was speculated that the function of *LFY* was suppressed or lost in ‘Kirakiraboshi.’ *LFY* and its homolog *FLO* have been identified in many plants such as *Arabidopsis thaliana* and *Antirrhinum majus*, and are known as transcription factors for major flowering signals.^30–32^ Additionally, many phenotypes of *Arabidopsis lfy* mutants have been reported.^32^^,^^33^ In the *lfy* strong phenotype, most organs are sepal-like, or mosaic sepal/carpel organs and the sepal-like organs are characteristic of wild-type cauline leaves.^34^

A similar phenotype has been reported in the *LFY* homolog mutants or transgenic plants such as the *flo* mutant of *Antirrhinum majus*,^35^*uni* mutant of pea,^36^ and co-suppressed *NFL* transgenic tobacco.^37^ As stated above, when the *LFY* gene function is lost, petals, stamens, and carpels are likely to be replaced by sepal-like organs. In addition, when male sterility was observed in the *Arabidopsis lfy* mutant, female fertility was reduced due to the lack of stamens, but remained due to the persistence of the carpel.^34^ In the decorative flowers of hydrangea, sepals show petaloid characteristics, including pigmentation and enlarged organ size.^1^ In addition, the double flower derived from *d_su_* showed male sterility and reduced female fertility. These phenotypes in double flower hydrangea are similar to *lfy* mutants. Thus, it is reasonable to assume that the double flower phenotype of *d_su_* is derived from the transformation of petals and stamens into sepal-like organs caused by an *LFY* mutation. We assumed that *LFY* is a causative gene of the double flower phenotype of ‘Sumidanohanabi.’

However, there remain several unexplained observations in this study. The double flower of ‘Kirakiraboshi’ did not exhibit the same phenotype as the *lfy* mutant. Generally, the flowers of *lfy* or its orthologous gene mutants have only leaf-like or sepal-like organs that have chlorophyll, stomata, and trichomes, and these organs have almost no petal identity.^34^^,^^35^ When flowering signals in *lfy* mutants were completely lost, floral organs were not fully formed.^34–36^ It has also been reported that *lfy* mutants with an intermediate or weak phenotype sometimes develop petaloid organs.^34^ In the double flowers of ‘Kirakiraboshi,’ the floral organs keep their petal identity, have papilla cells, and are pink or blue. These phenotypes of ‘Kirakiraboshi’ might reflect partially remnant *LFY* function.

It should be considered that another *LFY* gene possibly exists in the genome, and it partially compensates for the LFY function. However, according to the genomic and Iso-Seq sequences of *H. macrophylla*, no other *LFY* gene was observed. The mechanism of keeping petal identity in the floral organ of the double flower cultivar is still unknown. Comparing gene expression of *LFY* and *LFY* regulated genes between double and single flower cultivars might reveal aspects of the mechanism.

### 3.8. Candidate causative gene for *d_jo_*

While *LFY* was found as a candidate gene for controlling the double flower phenotype of *d_su_*, we could not find any candidate gene for the *d_jo_* locus. One possible reason was that SNPs were not called in scaffold with the causative gene. In pseudomolecules, about half of the total length of the scaffold was not included since relevant SNPs were not called. Improvement of SNP density would be effective for discovering additional scaffolds that are tightly linked to *d_jo_*. Although a candidate gene for *d_jo_* could not be identified from the completely co-segregated scaffold sequencing, we predicted several candidate genes based on gene function.

Because the phenotype of the *d_jo_* mutant is very similar to the *d_su_* mutant, the *d_jo_* causative gene may be a gene associated with *LFY*, which was suggested as a candidate causative gene for the *d_su_* mutant. The absence of petals and stamens in *lfy* mutant flowers in *Arabidopsis* has been traced back to a failure to activate the petal- and stamen-specific B-class genes *APETALA3* (*AP3*) and *PISTILLATA* (*PI*).^34^ For activation of gene expression of *AP3*, *UNUSUAL FLORAL ORGANS* (*UFO*) is required as a transcriptional cofactor of *LFY*.^38^ It is reported that the *ufo* mutant showed a similar phenotype with the *lfy* mutant in *Arabidopsis*.^39^ According to the regulatory network, the mutation in *UFO* or B-class genes, could cause an increase in sepals and lack of stamens and petals.

Previously, *HmPI*, *HmAP3*, and *HmTM6* were identified as B-class genes in hydrangea.^40^^,^^41^ As *HmAP3* was located on CHR13 as 0773 F.1_g216110, it was not considered as a causative gene for *d_jo_*. Although *HmPI* was found as 0880 F.1_g231800 and *HmTM6* as 0266 F.1_g601980, they were not included in the pseudomolecule. Ascertaining the loci of these genes might reveal the causative gene for *d_jo_*. On the contrary to B-class genes, the *UFO* gene has not been reported in hydrangea. According to HMA_r1.2 scaffold sequences, the *UFO* gene was found as 0185 F.1g081460. Because this gene was located on pseudomolecule CHR17_ 53812239-53813558, which was adjacent to *d_jo_*, it should be a candidate gene for *d_jo_*. Although we could not find a loss of function mutation of *UFO* CDS in the *d_jo_* mutant cultivar ‘Posy Bouquet Grace’ (data are not shown), it is still possible that the *UFO* gene function is lost. One possibility is that the loss of gene expression occurs owing to large INDEL in the promoter region of *UFO*. Further genome sequence comparison could clarify these possibilities.

### 3.9. DNA marker development for *d_jo_* and *d_su_*

**Figure 5:**
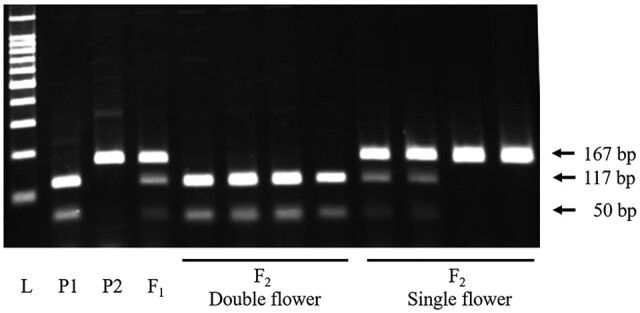
Fragment pattern of J01 DNA marker. Dominant single flower allele is shown as an undigested 167 bp fragment. Recessive double flower allele is shown as digested 117 and 50 bp fragments. L: 100 bp ladder, P1: ‘Posy Bouquet Grace’ (117_50/117_50), P2: ‘Blue Picotee Manaslu’ (167/167).

For the development of the CAPS marker linked to *d_jo_*, SNPs that were completely co-segregated with the double flower phenotype were selected. As a result, the CAPS marker J01 was successfully developed based on the SNP at scaffold 0008F-2_780104. The J01 CAPS marker amplified 167 bp of the fragment by PCR, and digestion with *Taq* I restriction enzyme generated 50 and 117 bp fragments in the double flower allele (Fig. 5). The J01 marker showed a high degree of agreement with the flower phenotype at 99.0% in the 15IJP1 and 100% in the 14GT77 populations that segregated the double flower phenotype of ‘Jogasaki’ (Supplementary Tables S4 and S5). This indicated that the J01 marker was tightly linked to the *d_jo_* locus, and J01 could effectively distinguish the recessive allele from the dominant allele.

**Figure 6:**
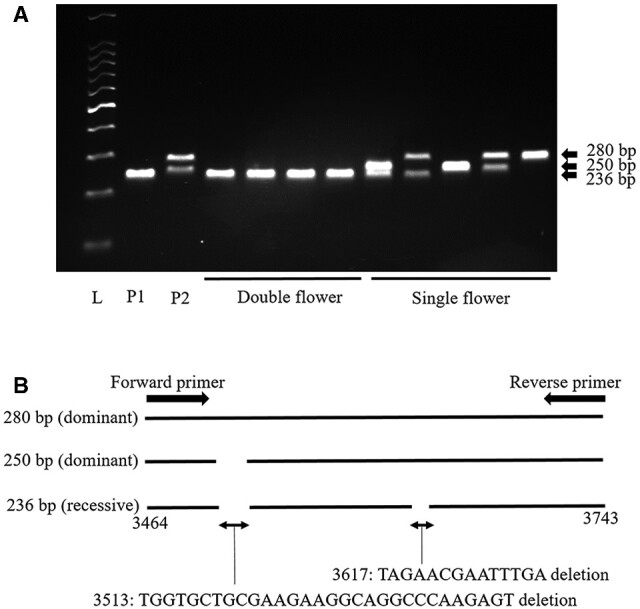
Fragment pattern of S01 DNA marker and INDEL polymorphism of amplified sequences. A. Fragment pattern of S01 DNA marker. Dominant alleles for *d_su_* are shown as 250 bp and 280 bp fragments. Recessive allele for dsu is shown as 236 bp fragments. L: 100 bp ladder, P1: ‘Kirakiraboshi’ (236/236), P2: ‘Frau Yoshimi’ (250/280). B. INDEL polymorphisms in alleles of DNA marker S01 amplified sequences. Positions on schematic models are the same as in Fig. 3.

For the development of the DNA marker for *d_su_*, an INDEL marker named S01 was developed on the *LFY* gene. To develop a DNA marker for distinguishing the *d_su_* recessive allele from the dominant alleles in the *LFY* genomic sequence, we focused and designed a DNA marker on the ‘Kirakiraboshi’ specific 14 bp deletion at position 3,617 from the initiation codon (Fig. 3A). We developed an S01 marker amplified 236 bp fragment for the double flower allele of ‘Kirakiraboshi,’ and 250 bp and 280 bp fragments for the single flower allele of ‘Frau Yoshimi’ (Fig. 6A). Three types of alleles resulted from the presence or absence of a 30 bp deletion at position 3,513 in addition to the 14 bp deletion. These were 30 bp and 14 bp deletions on the 236 bp allele, 30 bp deletion on the 250 bp allele, and no deletion on the 280 bp allele (Fig. 6B).

### 3.10. DNA marker application for hydrangea accessions

Since the J01 marker could distinguish *d_jo_* alleles and the S01 marker could distinguish *d_su_* alleles, the combined use of J01 and S01 DNA markers was expected to reveal the origin of the double flower phenotype, *d_jo_* or *d_su_*, in various accessions. Therefore, we performed DNA marker genotyping on *H. macrophylla* accessions using two DNA markers, J01 and S01. All tested double flower accessions showed homozygous genotypes of J01 or S01; 10 of the double flower accessions were homozygous of 117_50 in J01, and 4 were homozygous of 236 in S01 (Table 3). Contrarily, all single flower accessions showed other genotypes. Therefore, developed DNA markers J01 and S01 could successfully identify recessive double flower alleles for *d_jo_* and *d_su_*, respectively. Both markers showed a high degree of agreement with phenotype and were applicable to the examined *H. macrophylla* accessions. These DNA markers could be useful in marker-assisted selection (MAS) of double flower progenies. Identification of flower phenotype at the seedling stage by MAS would enable the discarding of single flower individuals and allow the growth of double flower individuals. The developed DNA markers should accelerate the breeding of double flower phenotypes.

**Table 3:** Genotypes of DNA markers J01 and S01 in *H. macrophylla* accessions

Accession name	Phenotype	Genotype	
		J01	S01
Jogasaki	Double	117_50/117_50	250/280
Posy Bouquet Grace	Double	117_50/117_50	280/280
Izunohana	Double	117_50/117_50	250/280
Chikushinokaze	Double	117_50/117_50	250/280
Chikushinomai	Double	117_50/117_50	280/280
Chikushiruby	Double	117_50/117_50	280/280
Corsage	Double	117_50/117_50	280/280
Dance Party	Double	117_50/117_50	280/280
Fairy Eye	Double	117_50/117_50	250/280
Posy Bouquet Casey	Double	117_50/117_50	250/280
Sumidanohanabi	Double	167/167	236/236
Kirakiraboshi	Double	167/167	236/236
HK01	Double	167/167	236/236
HK02	Double	167/167	236/236
03JP1	Single	117_50/167	280/280
Amethyst	Single	167/167	250/280
Aogashima-1	Single	167/167	280/280
Blue Picotee Manaslu	Single	167/167	280/280
Blue Sky	Single	167/167	280/280
Bodensee	Single	167/167	250/250
Chibori	Single	167/167	280/280
Furau Mariko	Single	167/167	250/250
Furau Yoshiko	Single	167/167	280/280
Furau Yoshimi	Single	167/167	250/280
Green Shadow	Single	167/167	280/280
Kanuma Blue	Single	167/167	250/280
Mrs. Kumiko	Single	167/167	280/280
Paris	Single	167/167	280/280
Peach Hime	Single	167/167	280/280
Picotee	Single	167/167	282/282
Ruby Red	Single	167/167	280/280
Shinkai	Single	167/167	280/280
Tokimeki	Single	167/167	280/282
Uzuajisai	Single	167/167	250/280

Genotypes shown in grey indicate homozygous of double flower allele.

## Supplementary data

Supplementary data are available at *DNARES* online.

## Supplementary Material

dsaa026_Supplementary_Data
