# Supplementary material for: Genome sequence of Hydrangea macrophylla and its application in analysis of the double flower phenotype
Source: DNA Res. 2020 Nov 11;28(1):dsaa026. doi: 10.1093/dnares/dsaa026 (PMC7934569; doi:10.1093/dnares/dsaa026)
Supplement: dsaa026_Supplementary_Data [file dsaa026_supplementary_data.zip › Supplementary Figure S1.pdf]

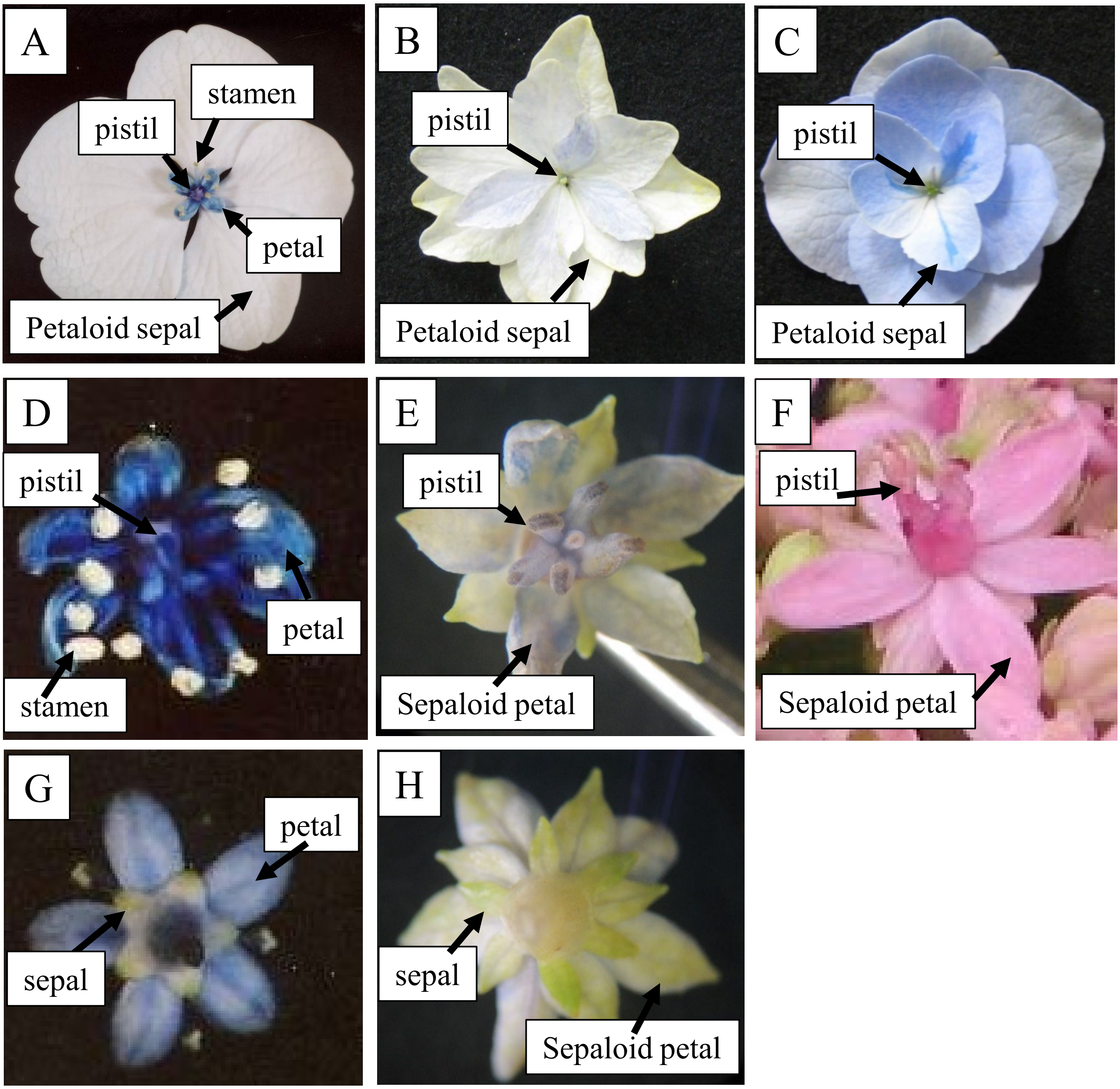

Supplementary Figure S1. Decorative and non-decorative flowers of single and double flower accessions

A-C: decorative flower. D-F: non-decorative flower. G, H: back side of non-decorative flower. A, D, G: Single flower cultivar ‘Lebelle.’ B, E, H: double flower cultivar ‘Sumindanohanabi.’ C, F: double flower cultivar ‘Jogasaki.’
