# Supplementary material for: Genome sequence of Hydrangea macrophylla and its application in analysis of the double flower phenotype
Source: DNA Res. 2020 Nov 11;28(1):dsaa026. doi: 10.1093/dnares/dsaa026 (PMC7934569; doi:10.1093/dnares/dsaa026)
Supplement: dsaa026_Supplementary_Data [file dsaa026_supplementary_data.zip › Supplementary Figure S3.pdf]

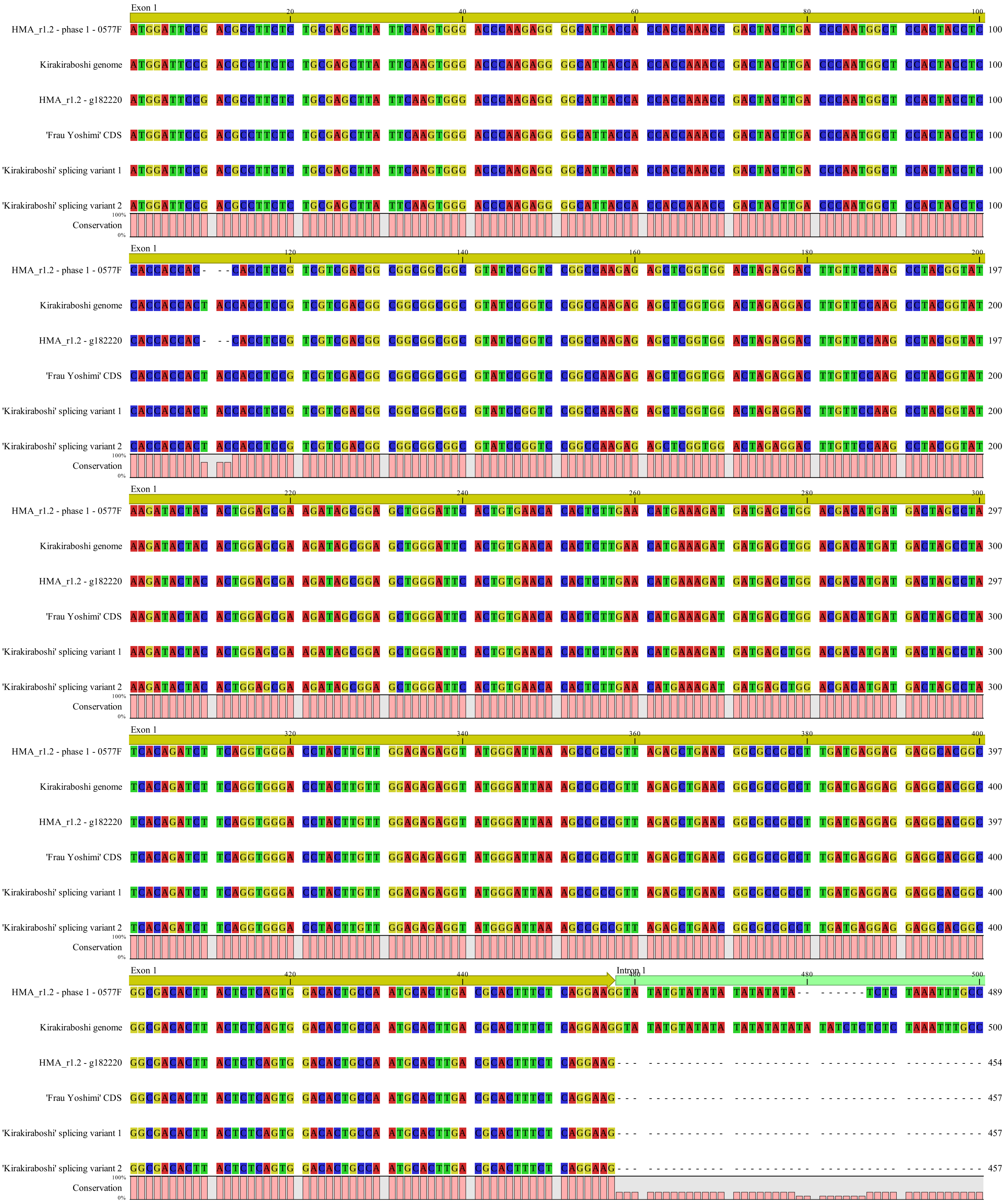

Supplementary Figure S3. Alignment of *LFY* genomic sequence and CDS.

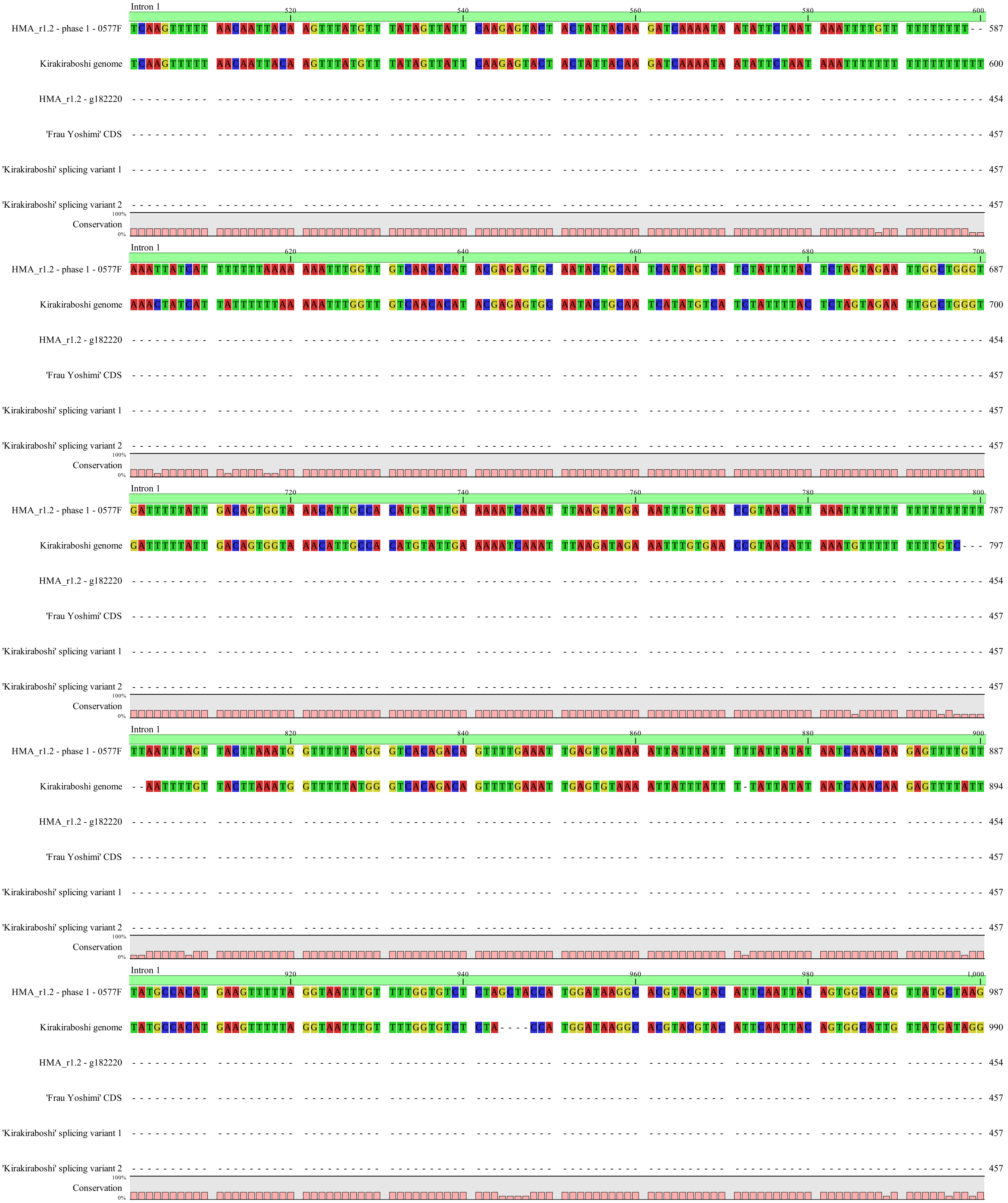

Supplementary Figure S3. Alignment of *LFY* genomic sequence and CDS. (continued)

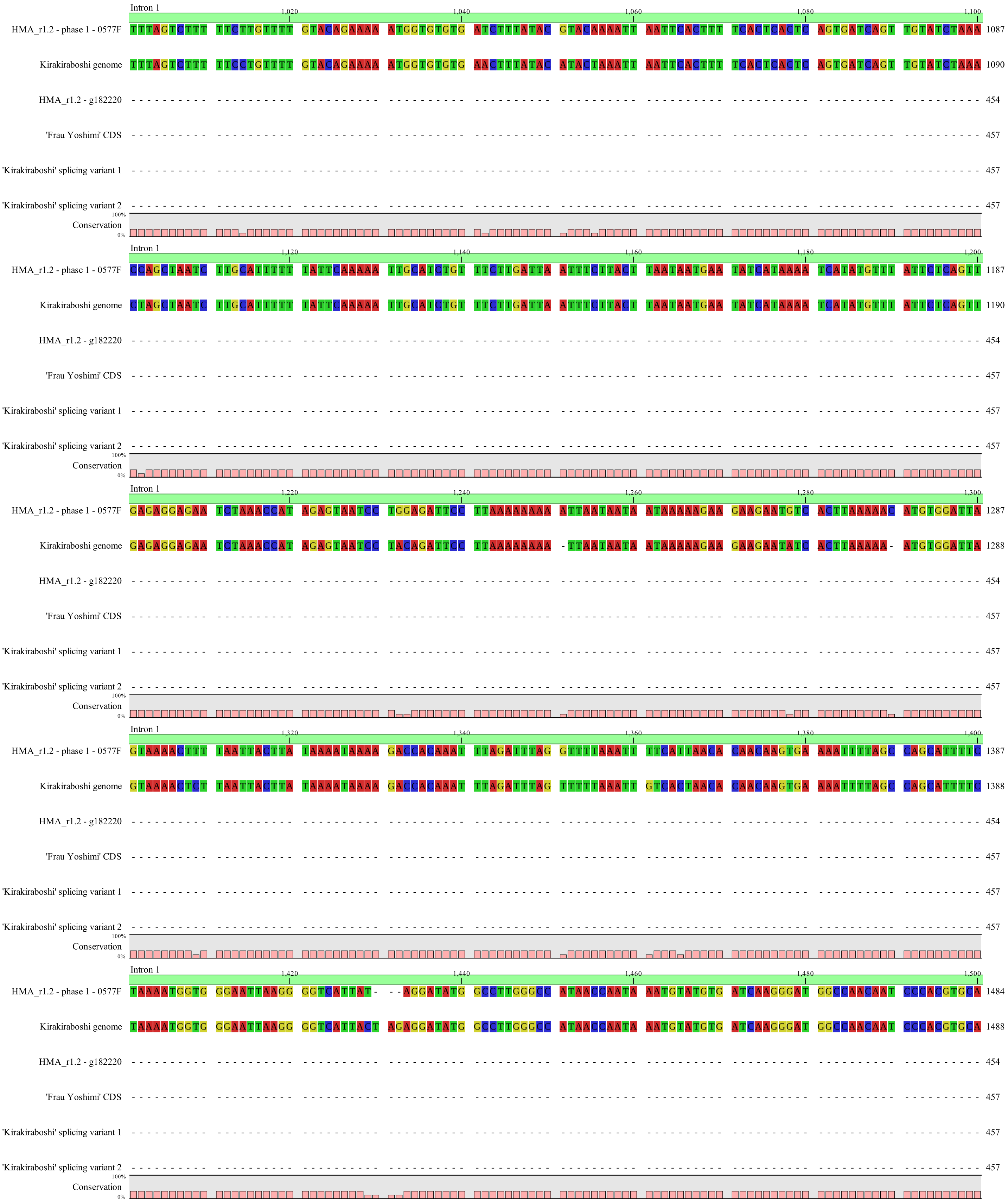

Supplementary Figure S3. Alignment of *LFY* genomic sequence and CDS. (continued)

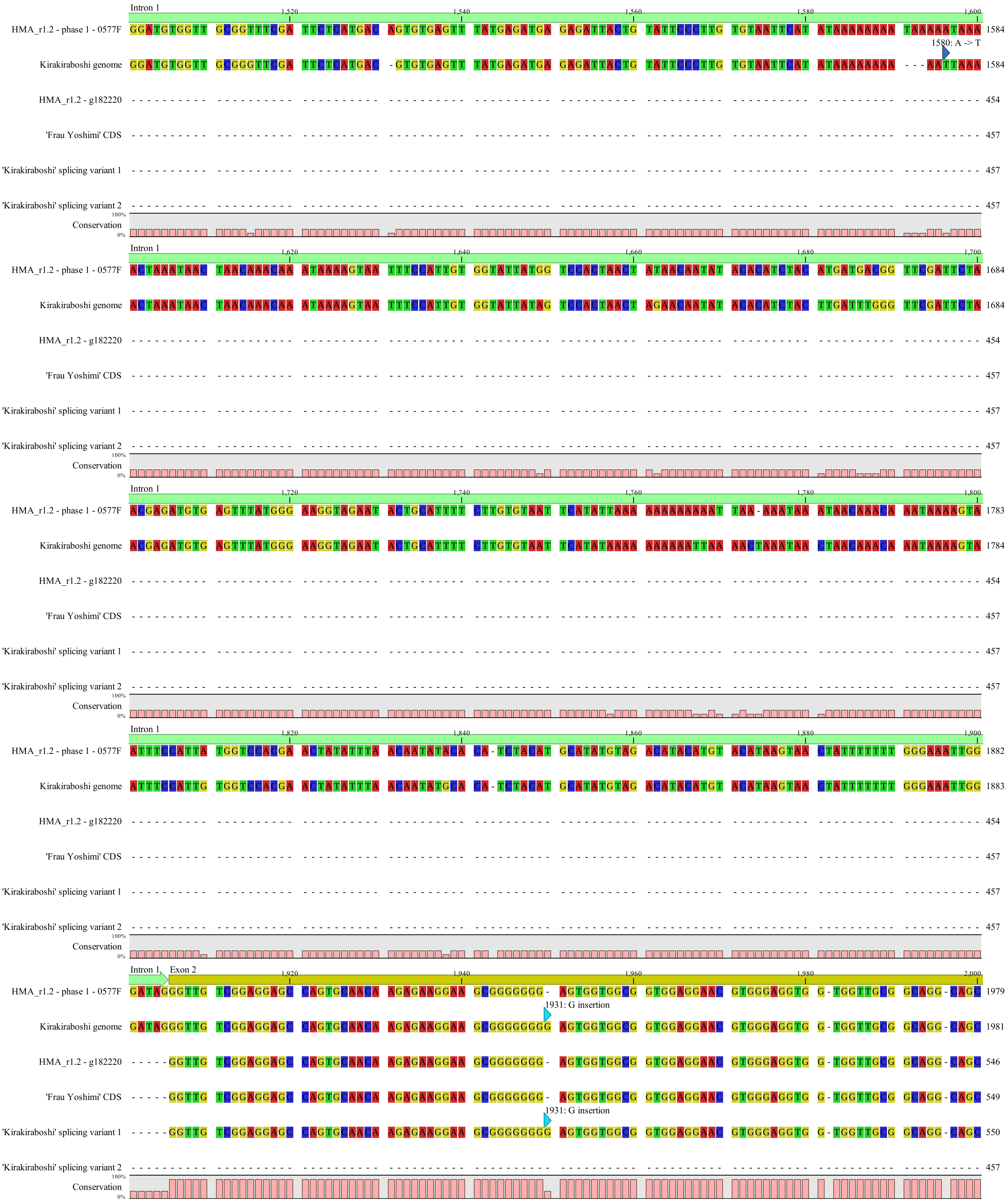

Supplementary Figure S3. Alignment of *LFY* genomic sequence and CDS. (continued)

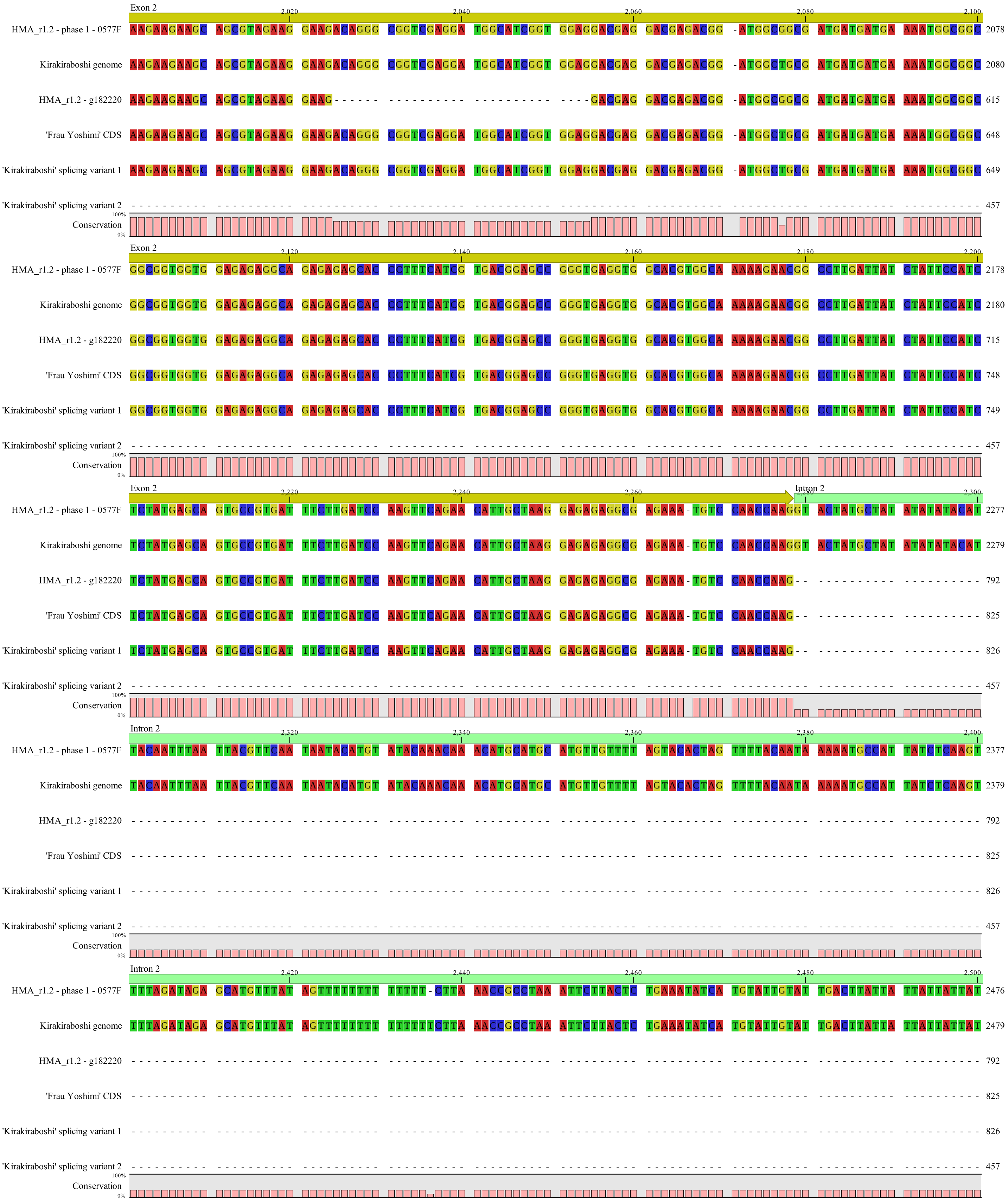

Supplementary Figure S3. Alignment of *LFY* genomic sequence and CDS. (continued)

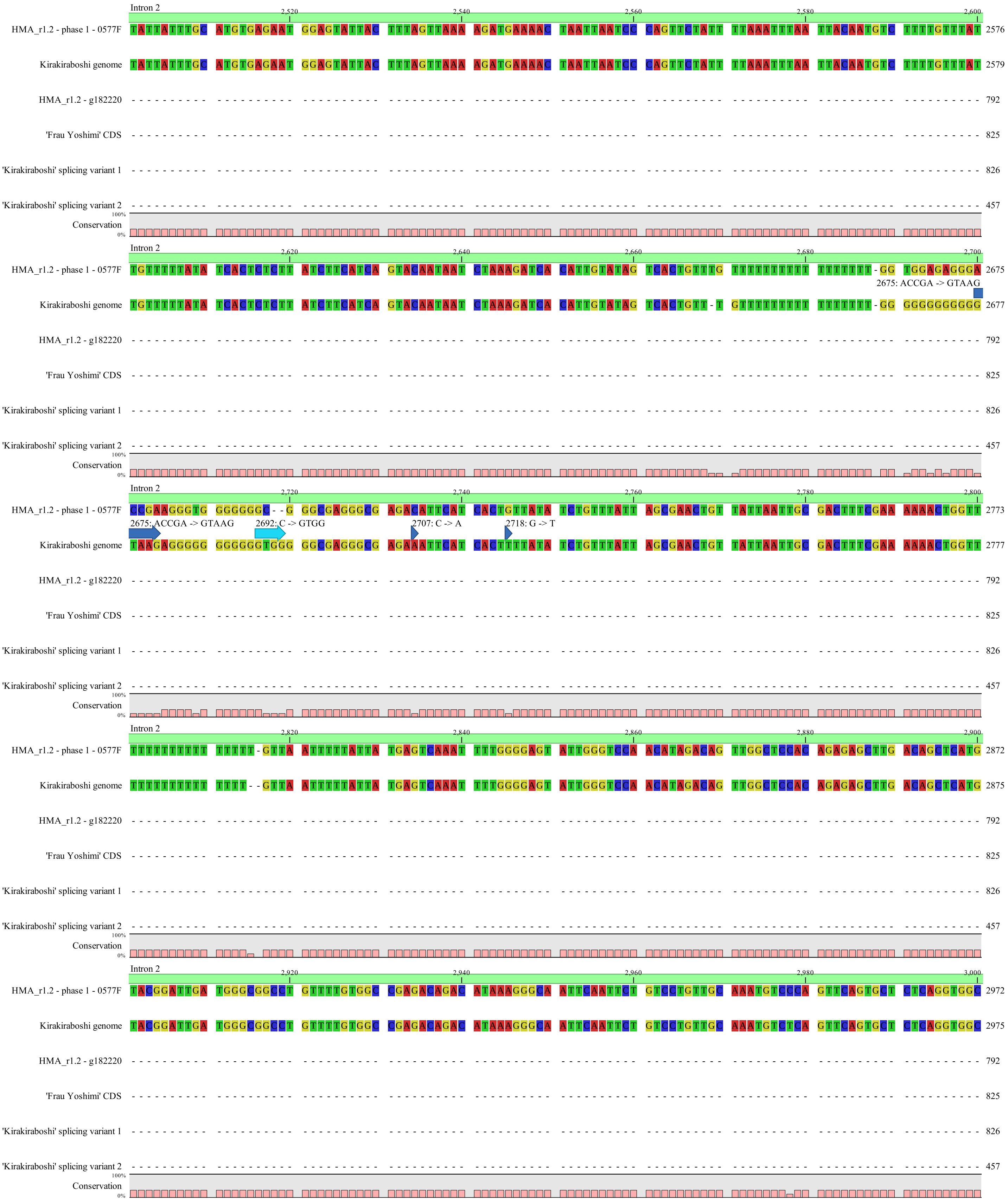

Supplementary Figure S3. Alignment of *LFY* genomic sequence and CDS. (continued)

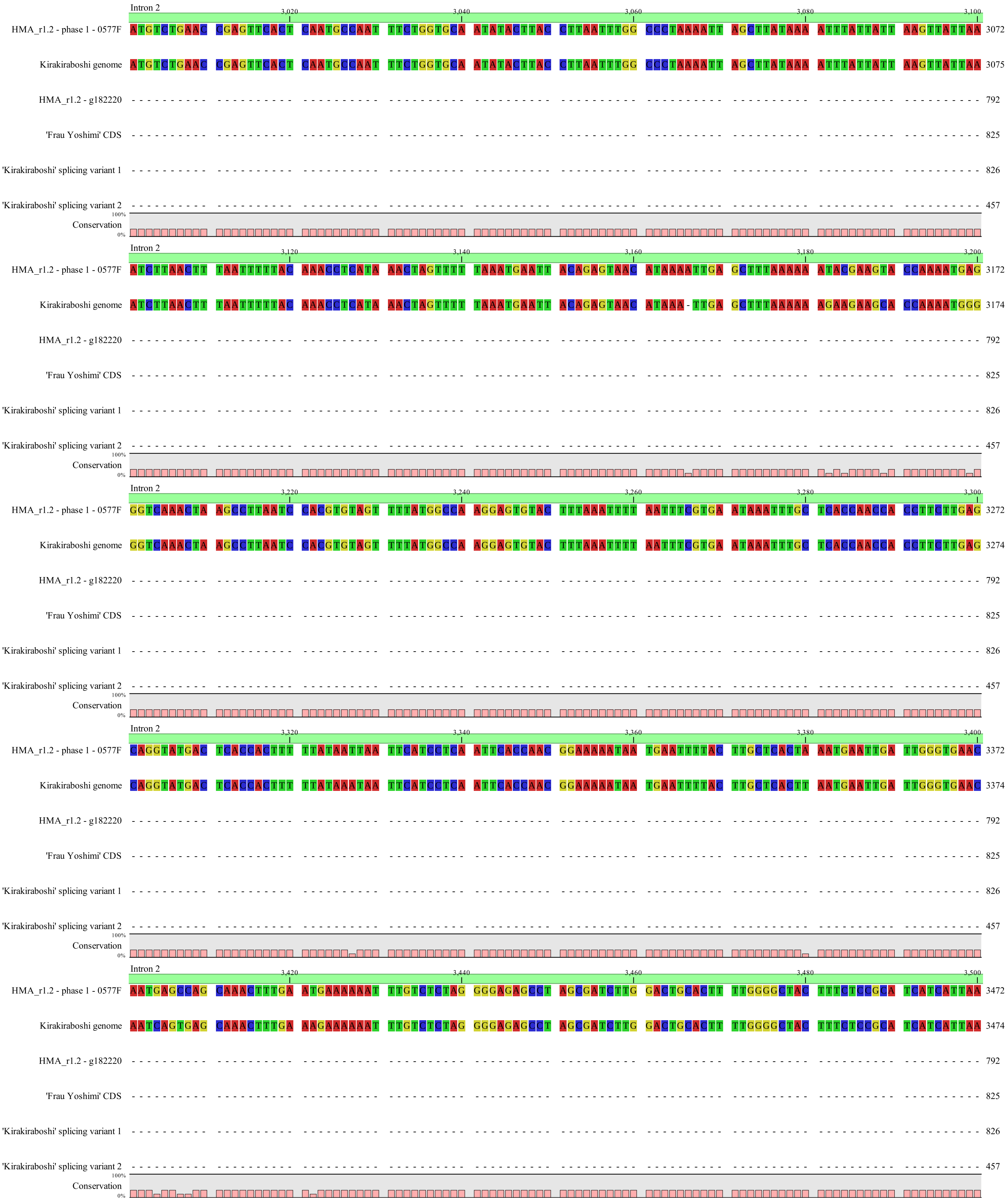

Supplementary Figure S3. Alignment of *LFY* genomic sequence and CDS. (continued)

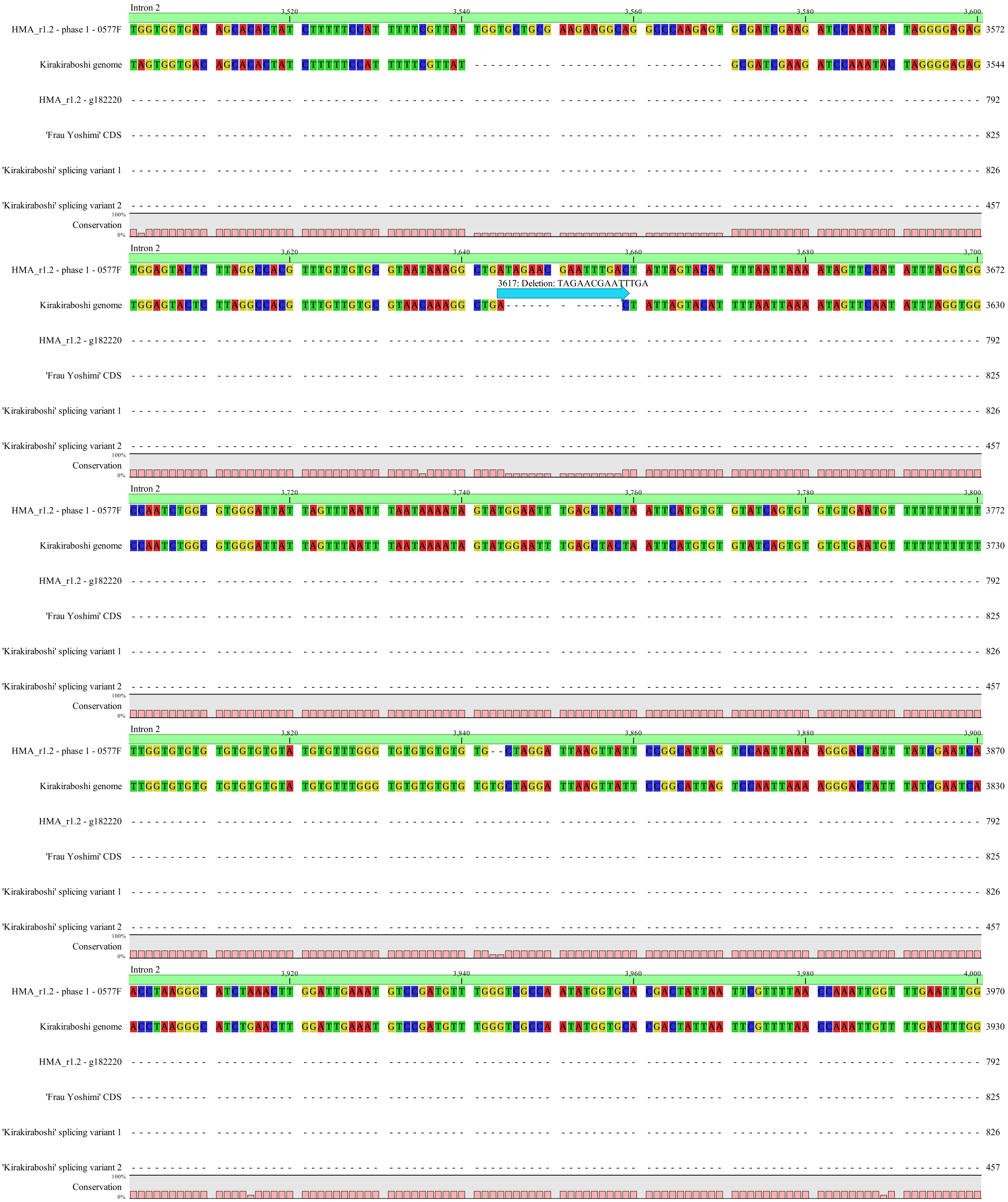

Supplementary Figure S3. Alignment of *LFY* genomic sequence and CDS. (continued)

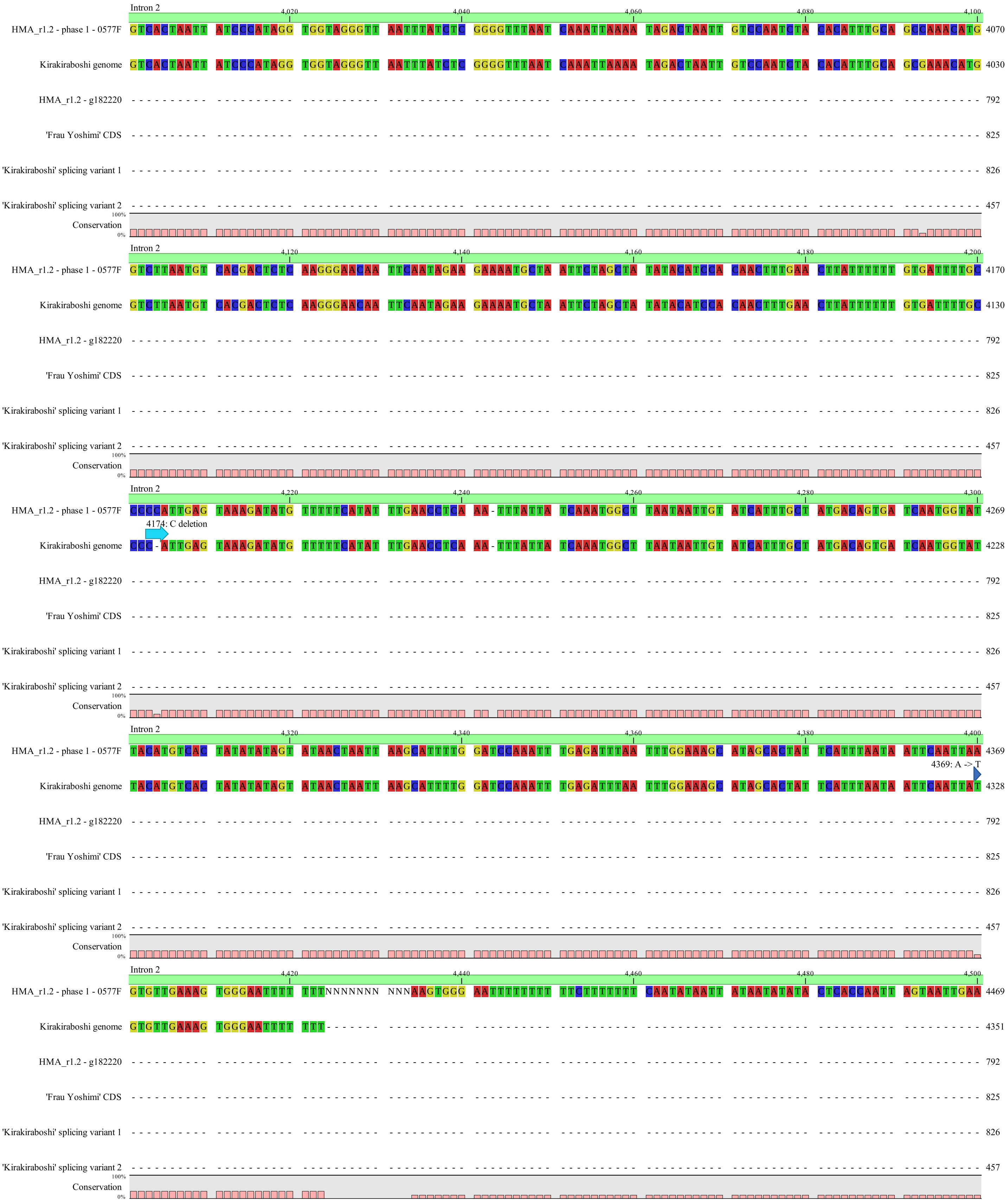

Supplementary Figure S3. Alignment of *LFY* genomic sequence and CDS. (continued)

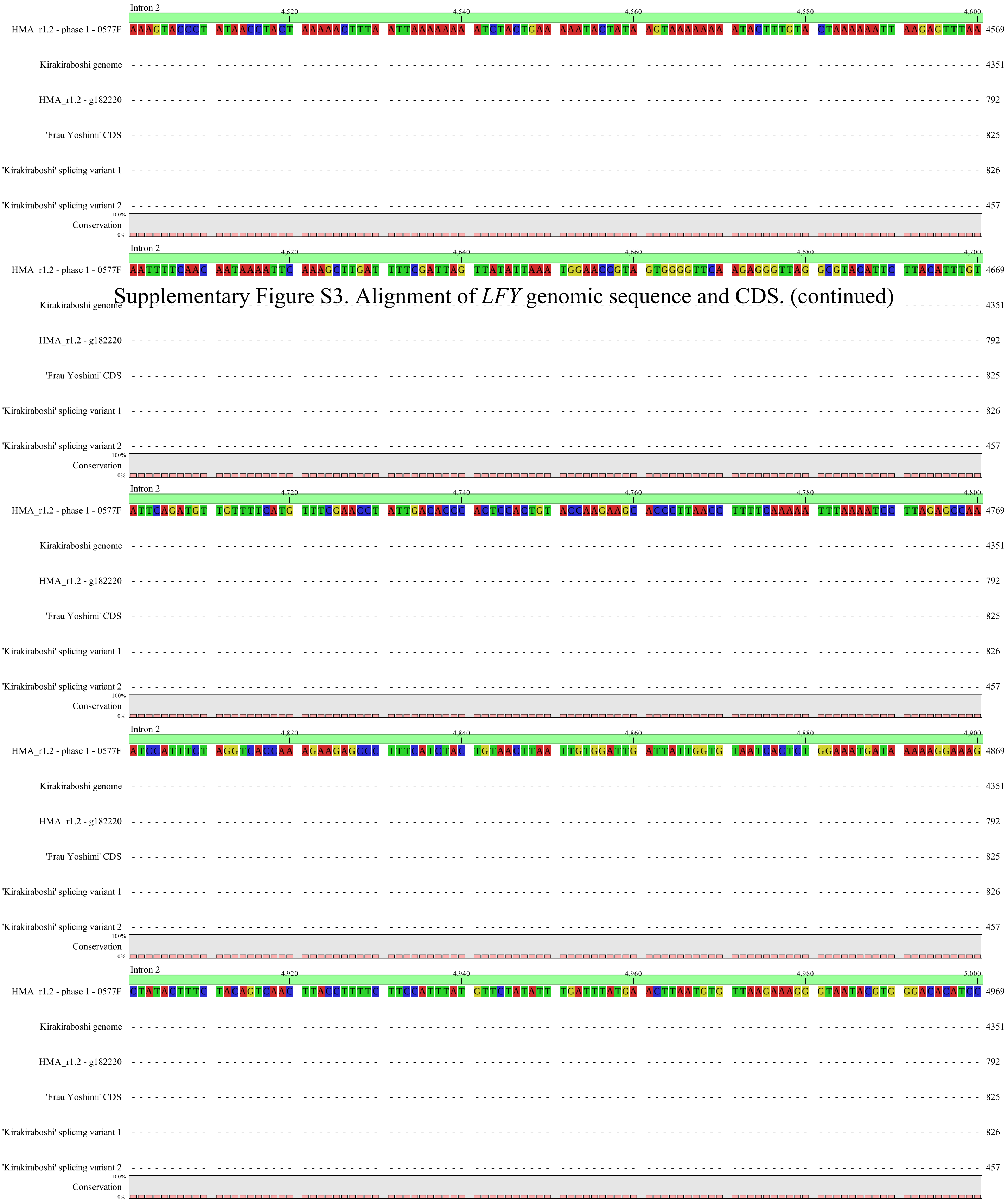

Supplementary Figure S3. Alignment of *LFY* genomic sequence and CDS. (continued)

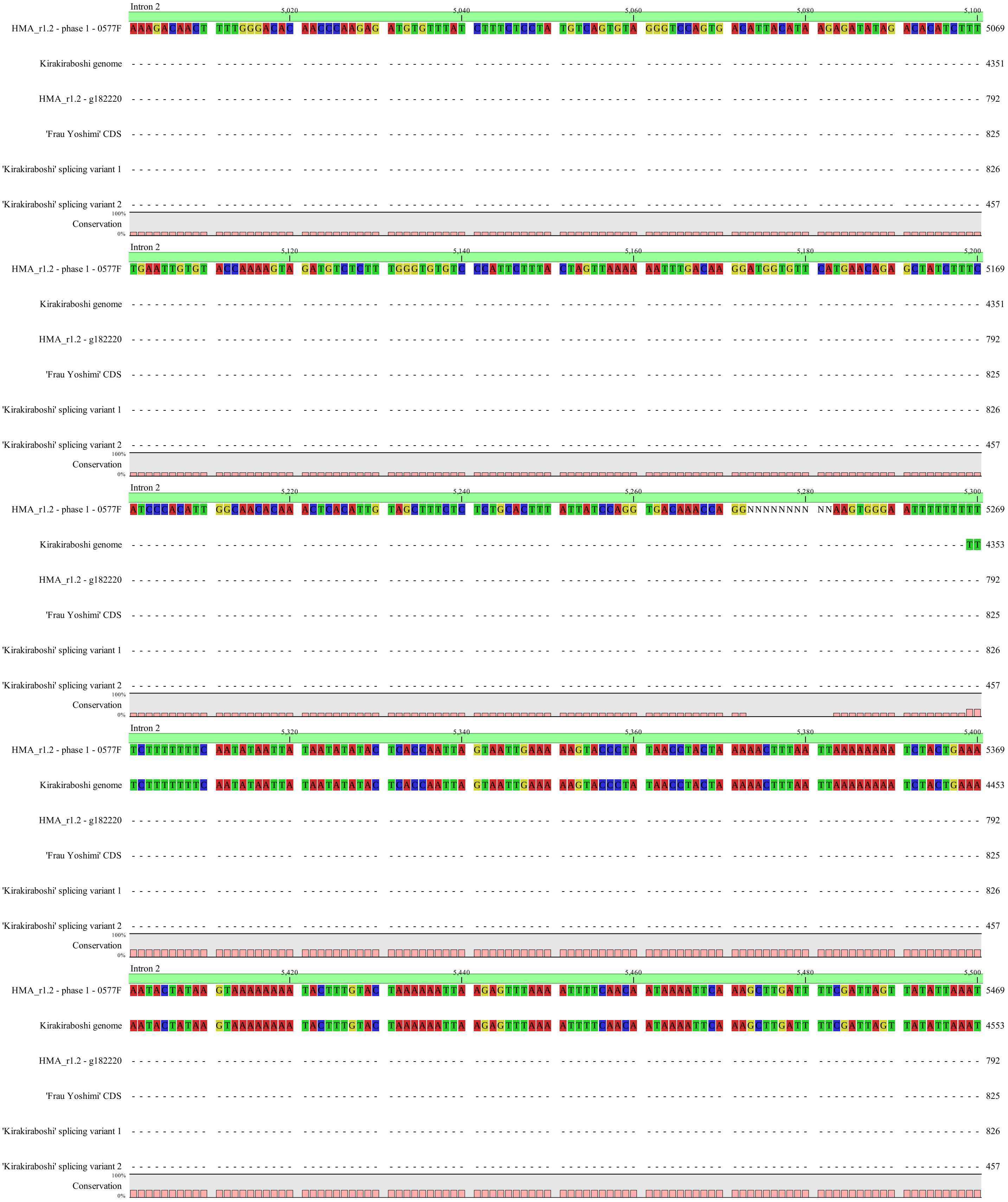

Supplementary Figure S3. Alignment of *LFY* genomic sequence and CDS. (continued)

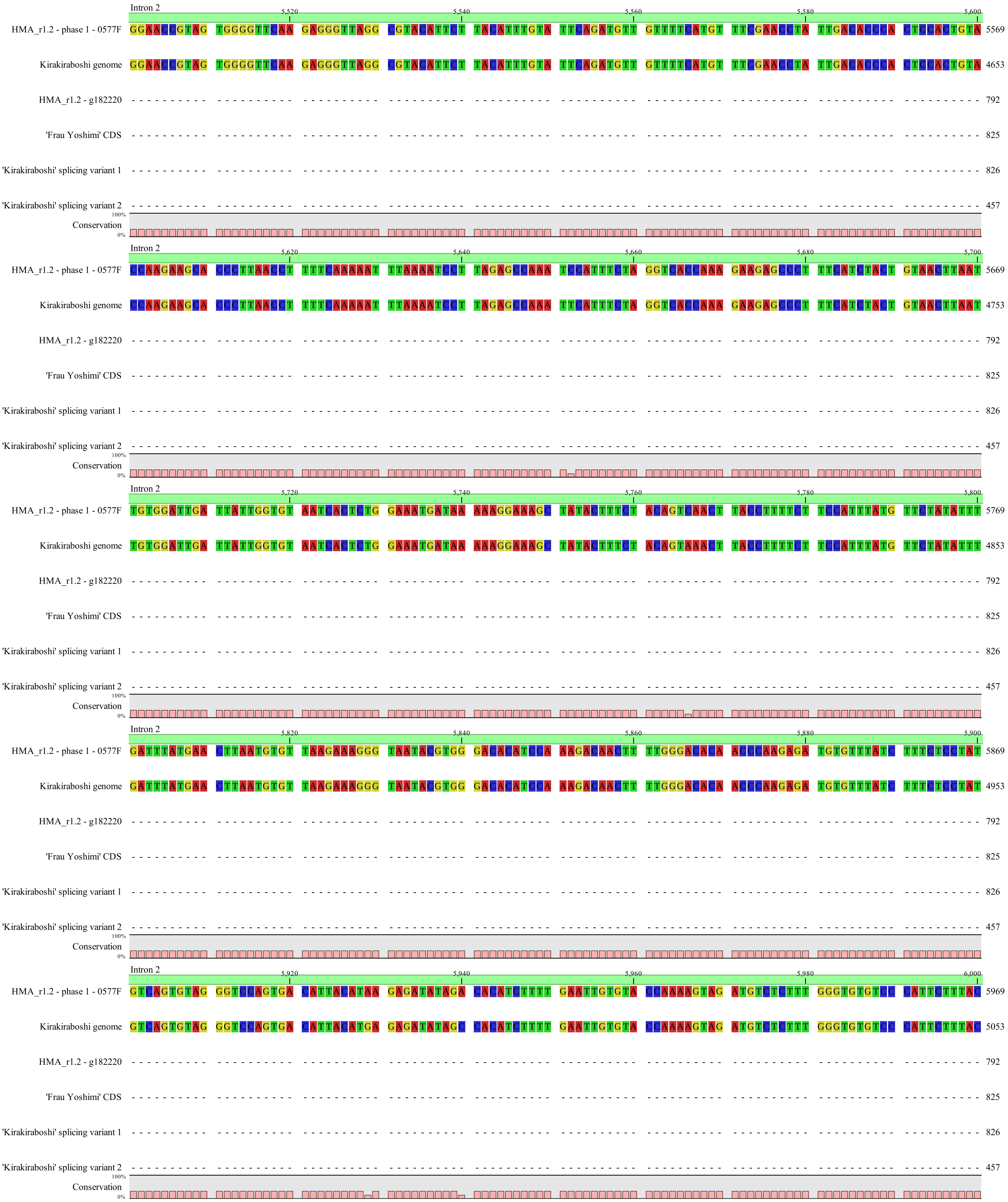

Supplementary Figure S3. Alignment of *LFY* genomic sequence and CDS. (continued)

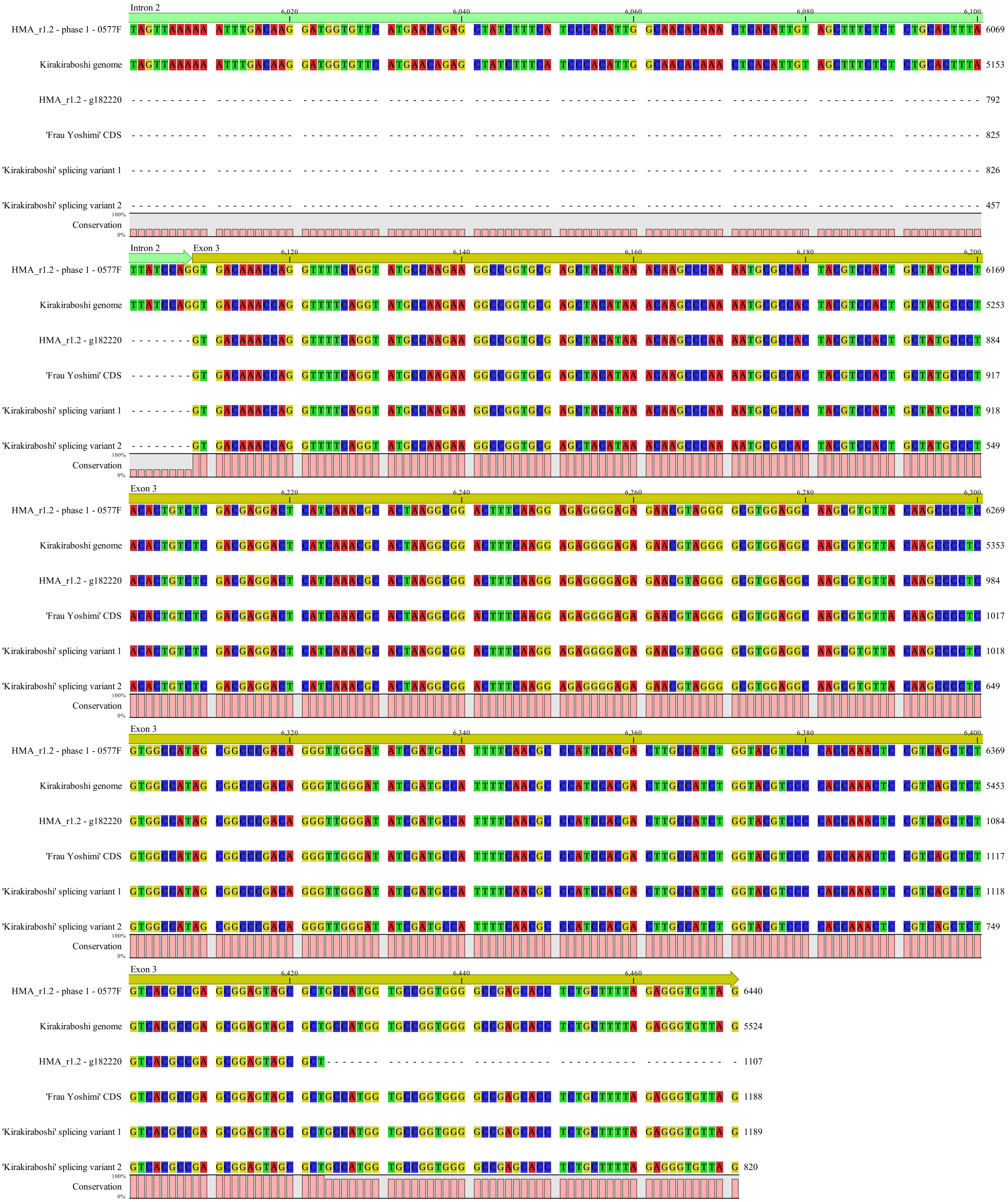

Supplementary Figure S3. Alignment of *LFY* genomic sequence and CDS. (continued)
