# Supplementary material for: Genome sequence of Hydrangea macrophylla and its application in analysis of the double flower phenotype
Source: DNA Res. 2020 Nov 11;28(1):dsaa026. doi: 10.1093/dnares/dsaa026 (PMC7934569; doi:10.1093/dnares/dsaa026)
Supplement: dsaa026_Supplementary_Data [file dsaa026_supplementary_data.zip › Supplementary_Table_S1.pdf]

Supplementary Table S1. RNA samples used for Iso-Seq and RNA-Seq

Sample No.	Accession	Species	Sampled organ	Sequencing method
1	Aogashima-1	*H. macrophylla*	Flower bud	Iso-Seq
2	Aogashima-1	*H. macrophylla*	Flower bud	Iso-Seq
3	Aogashima-1	*H. macrophylla*	Decorative flower	Iso-Seq
4	Aogashima-1	*H. macrophylla*	Decorative flower	Iso-Seq
5	Aogashima-1	*H. macrophylla*	Colored non-decorative flower	Iso-Seq
6	Aogashima-1	*H. macrophylla*	Colorless non-decorative flower	Iso-Seq
7	Aogashima-1	*H. macrophylla*	Fruit	Iso-Seq
8	Aogashima-1	*H. macrophylla*	Stem	Iso-Seq
9	Aogashima-1	*H. macrophylla*	Leaf bud	Iso-Seq
10	Aogashima-1	*H. macrophylla*	Leaf, one-day light-intercepted	Iso-Seq
11	Aogashima-1	*H. macrophylla*	Bud, one-day light-intercepted	Iso-Seq
12	Aogashima-1	*H. macrophylla*	Root	Iso-Seq
13	Blue Sky	Hybrid of *H. macrophylla* and *H. serrata var. yesoensis*	Flower bud	RNA-Seq
14	Spontaneous mutant of Blue Sky	Hybrid of *H. macrophylla* and *H. serrata var. yesoensis*	Flower bud	RNA-Seq
15	S-1	*H. macrophylla*	Flower bud	RNA-Seq
16	Spontaneous mutant of S-1	*H. macrophylla*	Flower bud	RNA-Seq
17	Jogasaki	*H. macrophylla*	Decorative flower	RNA-Seq
18	Sumidanohanabi	*H. macrophylla*	Decorative flower	RNA-Seq
19	Sumidanohanabi	*H. macrophylla*	Non-decorative flower	RNA-Seq
20	Sumidanohanabi	*H. macrophylla*	Non-decorative flower without petal	RNA-Seq
21	Wild hydrangea 1 (collected at Niijima, Tokyo, Japan)	*H. macrophylla*	Decorative flower	RNA-Seq
22	Wild hydrangea 1 (collected at Niijima, Tokyo, Japan)	*H. macrophylla*	Non-decorative flower	RNA-Seq
23	Wild hydrangea 1 (collected at Niijima, Tokyo, Japan)	*H. macrophylla*	Flower bud	RNA-Seq
24	Wild hydrangea 2 (collected at Tateyama, Chiba, Japan)	*H. macrophylla*	Leaf	RNA-Seq
25	Wild hydrangea 3 (collected at Izu-Oshima, Tokyo, Japan)	*H. macrophylla*	Flower bud	RNA-Seq
26	Wild hydrangea 4 (collected at Shirakawa village, Gihu, Japan)	*H. serrata*	Leaf	RNA-Seq
27	Hime Ajisai	Hybrid of *H. macrophylla* and *H. serrata var. yesoensis*	Flower bud	RNA-Seq
28	Hon Ajisai	*H. macrophylla*	Flower bud	RNA-Seq
29	Mixed accessions (13, 17, 20, 21, Leaf of 'Blue Sky' and non-decorative flower of 'Jogasaki')	*H. macrophylla* and *Hybrid of H. macrophylla and H. serrata var. yesoensis*	Leaf, non-decorative flower, decorative flower and flower bud	Iso-Seq
