# Supplementary material for: Genome sequence of Hydrangea macrophylla and its application in analysis of the double flower phenotype
Source: DNA Res. 2020 Nov 11;28(1):dsaa026. doi: 10.1093/dnares/dsaa026 (PMC7934569; doi:10.1093/dnares/dsaa026)
Supplement: dsaa026_Supplementary_Data [file dsaa026_supplementary_data.zip › Supplementary_Table_S2.pdf]

Supplementary Table S2 . Statistics of the genome sequences of *Hydrangea macrophylla* ‘Aogashima-1’

	HMA_r0.1	HMA_r1.0		HMA_r1.1		HMA_r1.2		HMA_r1.2.pmol	
		Primary contig	Haplotig	Phase 0	Phase 1	Phase 0	Phase 1	Phase 0	Phase 1
Total sequences	613,685	3,779	12,012	3,779	3,779	3,780	3,780	18	18
Assembly size (%)	1,714,141,309	2,178,088,391	1,436,935,012	2,256,097,326	2,227,567,818	2,256,097,326	2,227,567,818	1,077,798,883	1,076,873,717
Sequence N50	9,127	1,400,606	184,090	1,478,677	1,440,438	1,478,677	1,440,438	65,768,558	62,239,254
Gap size (bp)	5,985,045	1,051,170	895,550	1,273,060	1,069,590	1,273,060	1,069,590	673,130	569,310
%Gap	0.3	>0.0	0.1	0.1	>0.0	0.1	>0.0	0.1	0.1
Complete BUSCO	72.2	87.3	n.a.	n.a.	n.a.	84.8	87.7	n.a.	n.a.
Single-copy	69.4	79.8	n.a.	n.a.	n.a.	76.5	79.0	n.a.	n.a.
Duplicated	2.8	7.5	n.a.	n.a.	n.a.	8.3	8.8	n.a.	n.a.
Fragmented BUSCO	12.2	2.7	n.a.	n.a.	n.a.	3.7	2.6	n.a.	n.a.
Missing BUSCO	15.7	10.0	n.a.	n.a.	n.a.	11.5	9.7	n.a.	n.a.
#Genes	n.a.	n.a.	n.a.	n.a.	n.a.	33,848	34,149	n.a.	n.a.

n.a.: not analyzed

HMA_r0.1: Genome assembly based on the Illumina short-read data

HMA_r1.0: Genome assembly based on the PacBio long-read data

HMA_r1.1: Haplotype-phased genome assembly based on the PhaseGenomics Hi-C data

HMA_r1.2: Misassembly solved haplotype-phased genome assembly

HMA_r1.2.pmol: Chromosome-scale pseudomolecule assembly
