# Supplementary material for: Genome sequence of Hydrangea macrophylla and its application in analysis of the double flower phenotype
Source: DNA Res. 2020 Nov 11;28(1):dsaa026. doi: 10.1093/dnares/dsaa026 (PMC7934569; doi:10.1093/dnares/dsaa026)
Supplement: dsaa026_Supplementary_Data [file dsaa026_supplementary_data.zip › Supplementary_Table_S3.pdf]

Supplememtary Table S3. Correspondense of pseudomolecule sequences HMA_r1.2.pmol with genetic linkage map constructed by Waki et al.[5]

Pseudomolecules in this study	Genetic linkage map by Waki et al.[5]
CHR01	KF_1
CHR02	KF_2
CHR03	KF_3
CHR04	KF_4
CHR05	KF_5
CHR06	KF_6
CHR07	KF_7
CHR08	KF_8
CHR09	KF_9
CHR10	not specified
CHR11	KF_11
CHR12	KF_12
CHR13	not specified
CHR14	KF_14
CHR15	KF_15
CHR16	KF_16
CHR17	not specified
CHR18	not specified
