# Supplementary material for: Genome sequence of Hydrangea macrophylla and its application in analysis of the double flower phenotype
Source: DNA Res. 2020 Nov 11;28(1):dsaa026. doi: 10.1093/dnares/dsaa026 (PMC7934569; doi:10.1093/dnares/dsaa026)
Supplement: dsaa026_Supplementary_Data [file dsaa026_supplementary_data.zip › Supplementary_Table_S4.pdf]

Supplementary Table S4. J01 marker genotypes and double flower phenotypes of 15IJP1 population.

Genotype	Phenotype	
	Double flower	Single flower
Homozygous of 117_50 allele	39	0
Heterozygous	1	58
Homozygous of 167 allele	0	0
